# MALMPS: A Machine Learning‐Based Metabolic Gene Prognostic Signature for Stratifying Clinical Outcomes and Molecular Heterogeneity in Stage II/III Colorectal Cancer

**DOI:** 10.1002/advs.202501333

**Published:** 2025-07-17

**Authors:** Hao Chen, Ze Wang, Chenglong Sun, Yang Zhong, Yuan Liu, Yikun Li, Tongchao Zhang, Yuan Zhang, Xingyu Zhu, Leping Li, Feifei Teng, Ming Lu, Wei Chong

**Affiliations:** ^1^ Clinical Epidemiology Unit Clinical Research Center of Shandong University Qilu Hospital of Shandong University Jinan 250012 China; ^2^ Department of Epidemiology and Health Statistics School of Public Health Cheeloo College of Medicine Shandong University Jinan Shandong 250012 China; ^3^ Shandong Blood Center Jinan Shandong 250013 China; ^4^ Key Laboratory for Applied Technology of Sophisticated Analytical Instruments of Shandong Province Shandong Analysis and Test Center Qilu University of Technology Shandong Academy of Sciences Shandong 250014 China; ^5^ Department of Gastroenterological Surgery, Shandong Provincial Hospital Shandong University Jinan Shandong 250021 China; ^6^ Department of Radiation Oncology Shandong Cancer Hospital and Institute Shandong First Medical University and Shandong Academy of Medical Sciences Jinan Shandong 250117 China; ^7^ Department of Gastrointestinal Surgery Key Laboratory of Engineering of Shandong Province Shandong Provincial Hospital Affiliated to Shandong First Medical University Medical Science and Technology Innovation Center Shandong First Medical University Shandong Academy of Medical Sciences Jinan Shandong 250117 China; ^8^ School of Life Science and Technology Shandong Second Medical University Weifang Shandong 261053 China

**Keywords:** colorectal cancer, machine learning, metabolic‐related gene, prognosis, tumor metabolism

## Abstract

Colorectal cancer (CRC) is a frequently lethal disease, with stage II/III CRC accounting for ≈70%. Metabolic reprogramming plays a pivotal role in deciphering cancer heterogeneity and progression. Here, 9 datasets and 83 machine learning algorithm combinations are leveraged‌ to develop the Machine Learning‐based Metabolic gene Prognostic Signature (MALMPS) model. The MALMPS model outperformed traditional clinical traits and molecular features in predicting prognosis for stage II/III CRC patients across training and validation datasets. COX7B, a key gene in MALMPS, is shown to promote CRC malignancy through multi‐omics analysis and in vitro assays. CRC patients are stratified into high‐ and low‐risk groups based on the median cutoff of MALMPS. Notably, the high‐risk subgroup exhibited poor prognosis, activated inflammation, and enriched carbohydrate, glycosaminoglycan, and lipid metabolism, with therapeutic potential for IGF‐1R and Wnt/β‐catenin inhibitor. In contrast, the low‐risk group displayed a TGF‐β pathway inactivating mutation and enriched in nucleotides, cofactors, and amino acids metabolism. Metabolite profiling in the in‐house SDCRC dataset validated the distinct metabolic alterations between the two groups. These findings indicate that MALMPS is a valuable instrument for predicting the recurrence risk of stage II/III colorectal cancer, particularly for identifying individuals at high risk.

## Graphical abstract

Chen et al. identified a metabolic gene prognostic signature with machine‐learning framework and assessed the prognostic value in independent CRC cohorts. The metabolic signature subtypes are characterized by specific metabolic and proteomic characteristics, and associated with distinct molecular pathway and therapeutic potential.

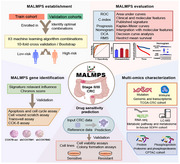

## 1 Introduction

Colorectal cancer (CRC) is the second‐most and third‐most common cancer in women and men, respectively. Moreover, it is still the second leading cause of death related to cancer, which causes a heavy disease burden.^[^ ^1^ ^]^ The TNM stage is a key prognostic factor for making therapy decisions, and stage II/III colorectal cancer accounts for ≈70% of all cases. However, despite advances in radiotherapy and chemotherapy, the overall survival rates for stage II/III colorectal cancer are still ≈70% and 50%, respectively.^[^ ^2^, ^3^ ^]^ The prognosis of stage II/III CRCs varies widely, and patients who routinely received adjuvant chemotherapy after surgery did not respond equally.^[^ ^4^ ^]^ These phenomena highlight the substantial heterogeneity among patients in stage II/III CRCs. The development of novel prognostic signatures for stage II/III CRCs represents a critical unmet need in clinical oncology.

Several studies have developed molecular models to predict stage CRC prognosis, including lncRNA, hallmark, and immune‐based signatures etc.^[^ ^5^, ^6^, ^7^, ^8^ ^]^ Although the prediction performances varied across different studies, there was a notable lack of emphasis on sufficient validation and exploration of diverse modeling algorithms. Metabolism reprogramming plays a significant role in the initiation, growth, and spread of CRC, and metabolic adaptations are closely linked to the pathways that lead to cancer.^[^ ^9^, ^10^ ^]^ Meanwhile, more refinement in modeling algorithm combination and signature validation is needed to improve the credibility of the model. With advances in sequencing technology, machine learning is increasingly being used to predict prognosis and patient survival based on genomic, metabolomic, and transcriptomic data.^[^ ^11^, ^12^ ^]^ Clinicians and researchers are now turning to machine learning techniques, such as neural networks, ensemble approaches, and deep learning, for patient outcomes classification and prediction.

Recent advancements in multi‐omics approaches, such as genomic, proteomic, metabolomic, scRNA‐seq, and spatial transcriptomics, have provided deeper insights into the molecular mechanisms underlying intratumoral heterogeneity and tumor‐associated cellular reprogramming.^[^ ^12^, ^13^ ^]^ Mass spectrometry imaging‐based spatially resolved metabolomics (SM) enables in situ screening of metabolic biomarkers associated with tumor initiation, progression, and metastasis, thereby facilitating the characterization of the metabolic architecture of tumors and their surrounding microenvironment.^[^ ^14^ ^]^

Under the background above, the study aims to screen metabolic‐related genes associated with clinical significance through a combined 10‐fold or bootstrap machine learning framework in stage II/III CRCs. The MAchine Learning‐based Metabolic Gene Prognostic Signature (MALMPS) model was validated in five other independent datasets. The biological activities, molecular mechanism, tumor microenvironment remodeling, metabolic reprogramming, and chemotherapy response across different MALMPS subgroups were further explored in multi‐omics approaches and in vitro assays. Finally, we established and validated a nomogram based on the integration of MALMPS and clinical and molecular features.

## 2 Results

### 2.1 Development of a Machine Learning‐Based Metabolic‐Related Gene Signature Model

**Figure 1:**
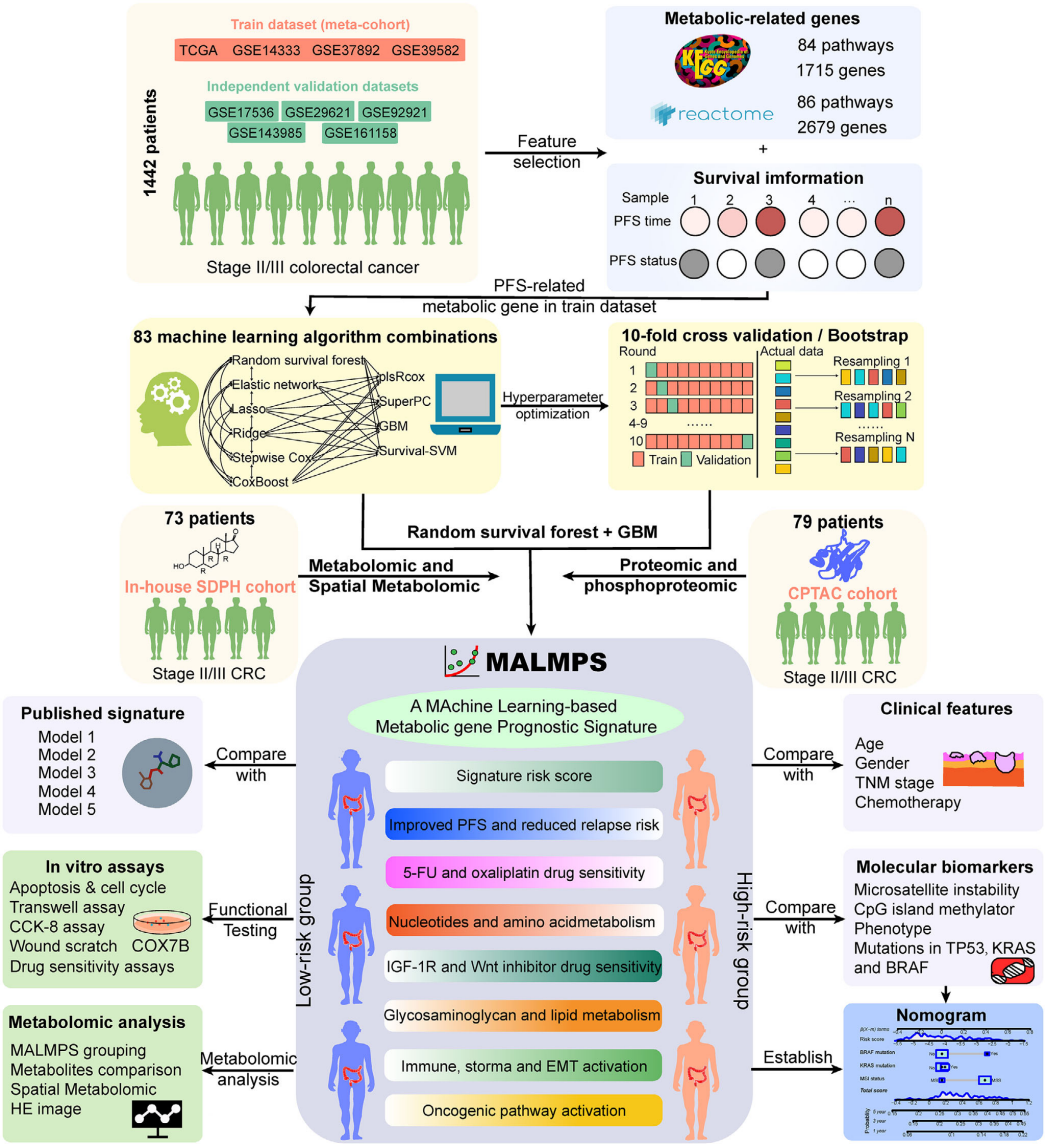
Overall workflow of the study.

**Figure 2:**
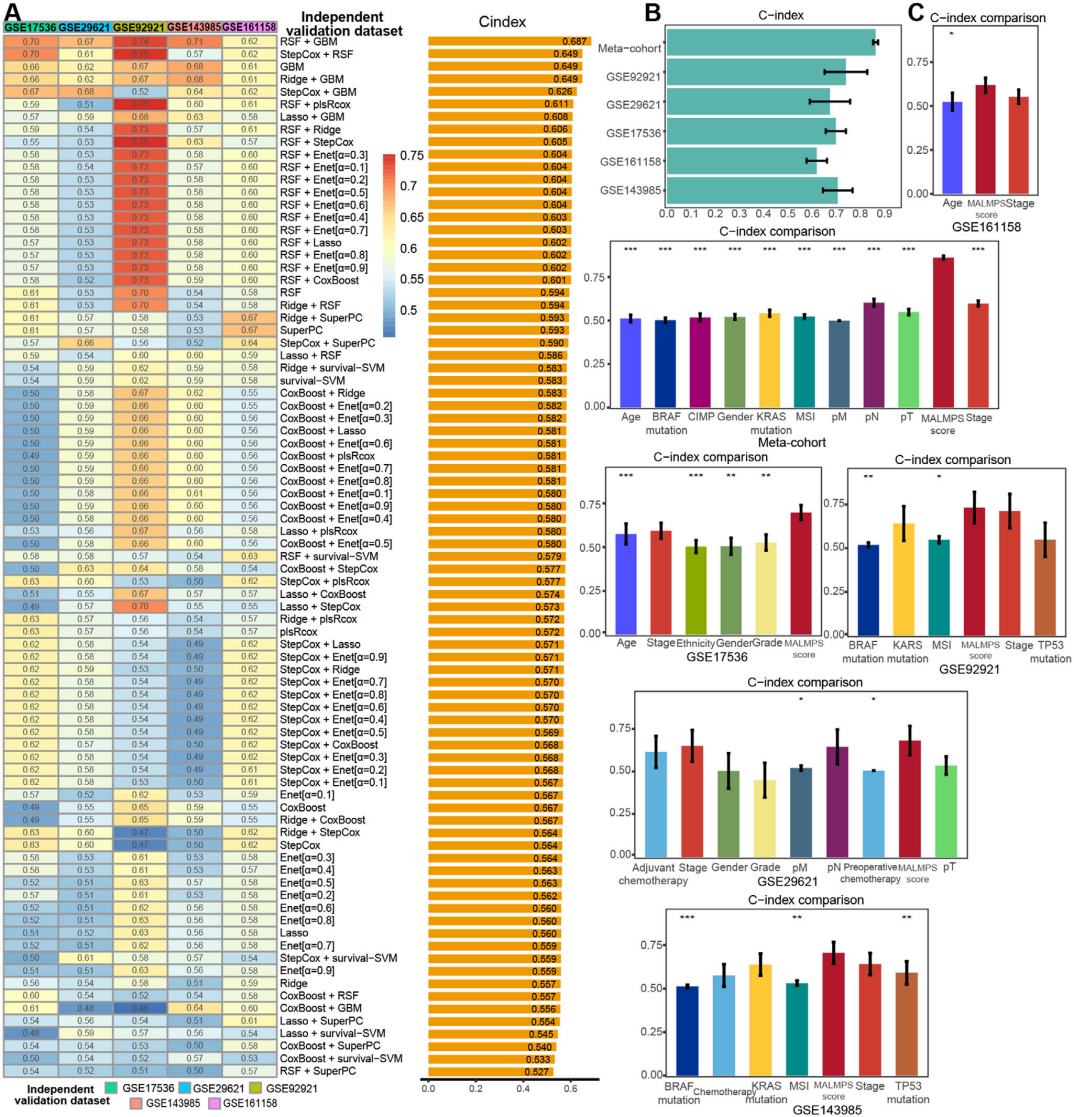
The identification of the best performance signature. A) C‐indices of 83 combinations of machine learning prediction models in five independent validation cohorts. B) C‐indices of MALMPS across all datasets. C) C‐index comparisons between clinical and molecular variables and MALMPS scores in GSE161158, training meta‐cohort, GSE17536, GSE92921, GSE29621, and GSE143985. * *P* < 0.05, ** *P* < 0.01, *** *P* < 0.001.

The overall study design workflow is presented in **Figure** **1**. A total of 1,053 overlapping genes, curated from the KEGG and REACTOME metabolic pathway, were subjected to univariate Cox regression analysis, which identified 149 metabolic‐related prognostic genes in the training meta‐cohort (Table S1, Supporting Information). The expression profiles of the genes were subjected to the machine learning‐based modeling framework. We fitted 83 kinds of prediction models based on 10‐fold CV or bootstrap resampling via ten machine learning algorithms and further calculated the C‐index of each model across all validation datasets. The most robust model with the highest mean C‐index in the five validation datasets was the combination of random survival forest (RSF) and generalized boosted regression modeling (GBM) (**Figure** **2**A), which showed a range of C‐index from 0.62 to 0.86 (training dataset: 0.862 [95%CI: 0.841‐0.882], GSE17536: 0.698 [95%CI: 0.635‐0.760], GSE29621: 0.673 [95%CI: 0.548‐0.798], GSE92921: 0.740 [95%CI: 0.607‐0.871], GSE143985: 0.705 [95%CI: 0.614‐0.797], GSE161158: 0.619 [95%CI: 0.556‐0.682], Figure 2B). In the identified model combination, we obtained the optimistic mtry and nodesize parameters with the lowest out‐of‐sample error under the number of ntree 845. The appropriate ntree number was picked according to the error rate plot, and features were selected according to the variable importance via parameter‐adjusted RSF analysis (Figure S1A, Supporting Information). The selected features were further subjected to GBM to fit the predictive model with the optimal number of iterations (Figure S1B, Supporting Information). Finally, the risk score for each patient was calculated using the expression of 21 gene features, weighted by the relative importance in the GBM model, to generate the MAchine Learning‐based Metabolic gene Prognostic Signature (MALMPS, Figure S1C and Table S2, Supporting Information) model. We compared the prognostic efficacy of the MALMPS with that of commonly used clinical and molecular features, including age, sex, ethnicity, AJCC stage, grade, as well as driver gene mutations (KRAS, TP53, and BRAF) and microsatellite instability (MSI), etc. Intriguingly, the MALMPS score consistently outperformed all other clinical and molecular features in terms of the C‐index across all independent validation datasets. All variables were statistically significant in the training dataset (*P* < 0.001), and some variables such as age, race, gender, BRAF mutation, and MSI status were statistically significant in the validation datasets (*P* < 0.05, *P* < 0.01, or *P* < 0.001), highlighting the superior diagnostic precision of our constructed signature (Figure 2C).

### 2.2 Evaluation of MALMPS Model Performance and Prognostic Value

**Figure 3:**
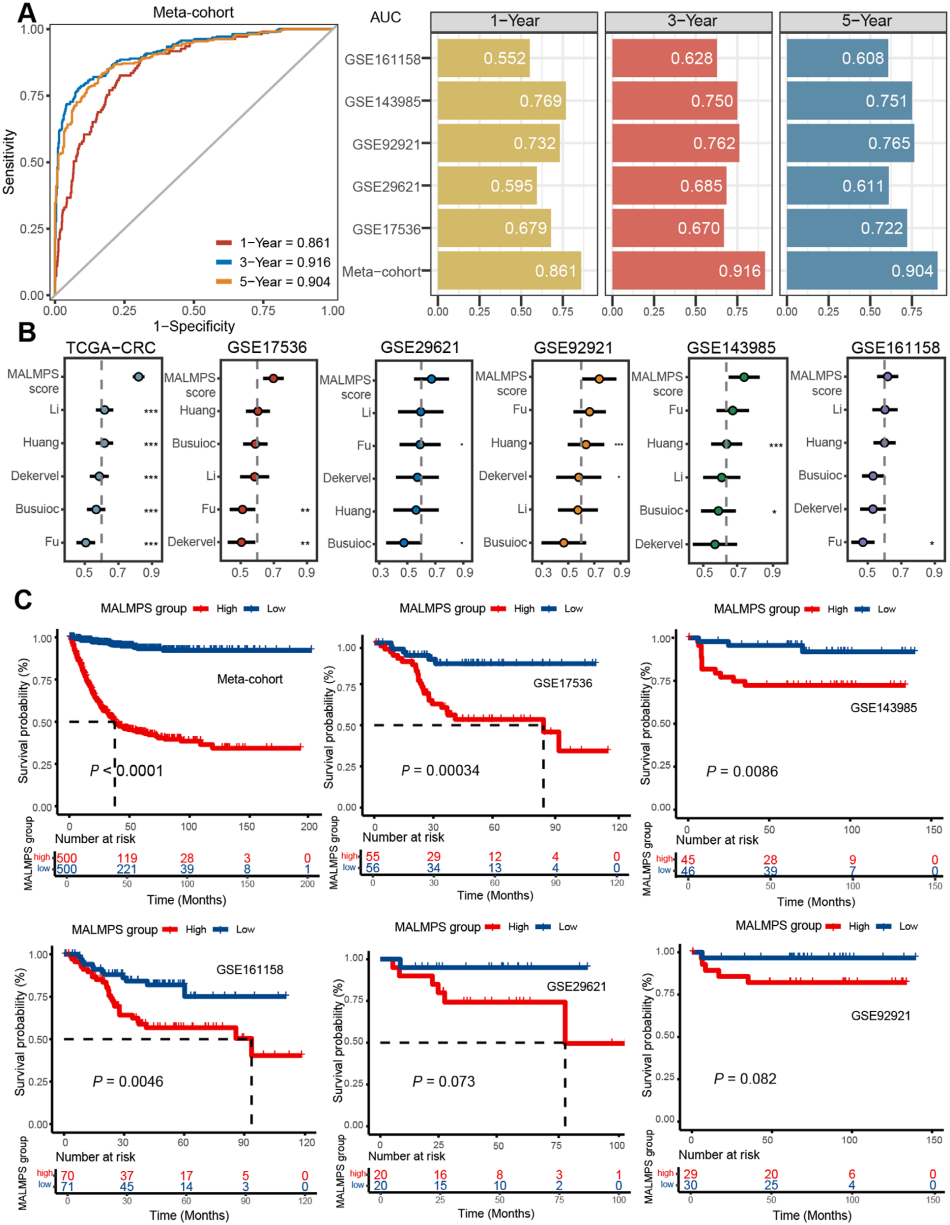
Evaluation of MALMPS performance. A) Time‐dependent ROC analysis for predicting PFS at 1, 3, and 5 years across the training meta‐cohort and all validation datasets. B) C‐index comparisons between MALMPS scores and 5 published signatures in TCGA‐CRC, GSE17536, GSE29621, GSE92921, GSE143985, and GSE161158. C) Kaplan‒Meier survival curve of PFS between patients with a high MALMPS score and those with a low MALMPS in all datasets. * *P* < 0.05, ** *P* < 0.01, *** *P* < 0.001.

We utilized the ROC analysis to measure the PFS discrimination of the MALMPS model (**Figure** **3**A). The average AUC for the training dataset was 0.893, and the model showed a consistently decent level of 3‐year AUCs across the validation datasets (average of 0.699). In addition, we systematically collected and re‐analyzed five prognosis molecular signatures in the CRC transcriptomic dataset (Table S3, Supporting Information). Our MALMPS scores achieved the highest C‐index among five published molecular signatures in the TCGA (training set) and additional independent validation datasets (Figure 3B). In summary, our MALMPS model could be a promising biomarker for predicting the PFS risk of stage II/III CRC patients.

The samples in our independent datasets were further stratified into high‐risk and low‐risk groups based on the median predicted MALMPS score. Patients in the high‐risk group had a significantly shorter PFS time than those in the low‐risk group in the training meta‐cohort (*P* < 0.0001), and similar trends were also observed in the validation datasets (*P *< 0.01 for three datasets, with GSE29621 and GSE92921 only marginally missing the statistical cut‐off, possibly due to their small sample sizes, with *P* values of 0.073 and 0.082, respectively. Figure 3C). The multifaceted evaluation demonstrated that the MALMPS model performed exceptionally well in identifying colorectal cancer patients with distinct clinical outcomes.

### 2.3 COX7B Promotes Malignant Behaviors in CRC

**Figure 4:**
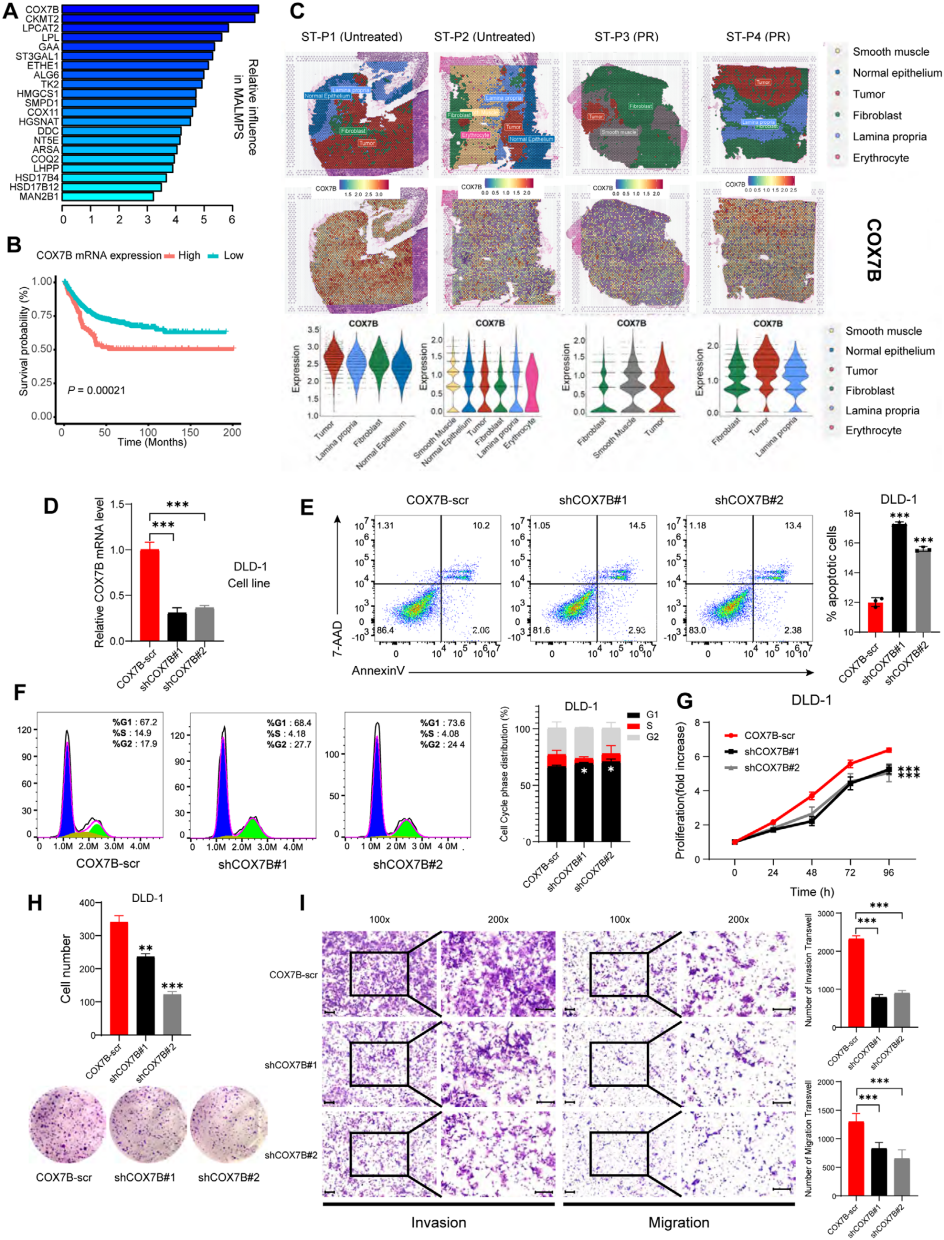
COX7B promotes the CRC malignant behaviors. A) The importance calculated by the GBM algorithm of the 21 most valuable metabolic genes determined by RSF algorithm. B) Kaplan‐Meier survival curve of PFS between patients with high COX7B and low COX7B mRNA expression. C) Heatmap and violin plot showing the unsupervised clustering analysis distribution and COX7B expression of ST among different cell types in CRC. D) Barplot showing the COX7B expression levels within COX7B‐scr, shCOX7B#1 and shCOX7B#2 groups. E) Scatter plots and barplot demonstrating the trends in the proportions of apoptotic cells. F) Histograms and barplot exhibiting the proportion of cells in different cell cycle phases. G) Line chart showing the proliferation fold increase within the three groups over time. H) Representative staining images and barplot of colony number among the three groups. I) Representative staining images and barplot of invasion and migration transwell number among the three groups. Scale bar, 100 µm. * *P* < 0.05, ** *P* < 0.01, *** *P* < 0.001.

COX7B emerged as the most relatively important gene in MALMPS, we further performed prognostic analysis and found that higher mRNA expression of COX7B was associated with worse PFS in stage II/III CRC patients (**Figure** **4**A,B). Spatial transcriptomic (ST) data of paired colorectal cancer and liver metastasis samples from two untreated patients and two neoadjuvant chemoradiotherapy patients showed that COX7B was highly expressed in tumor tissues both in colorectal and liver metastases (Figure 4C; Figure S2A, Supporting Information). We further analyzed the cell proliferation dynamics of the 21 genes in CRC cell lines based on Chronos, a model that infers gene fitness effects from CRISPR knockout screens. We observed that the Chronos score in COX7B knockout cell lines was all below zero, indicating its significance in the viability of tumor cells (Figure S2B, Supporting Information). Furthermore, proteomic analysis revealed that elevated COX7B protein levels were associated with poor prognosis in colorectal cancer (CRC) and correlated with increased levels of the tumor marker CA19‐9 (Figure S2C,D, Supporting Information). We then performed KEGG pathway enrichment analysis, which revealed that high COX7B expression is associated with key metabolic and cellular processes, including glycolysis, pyruvate, fatty acid metabolism, and the cell cycle (Figure S2E, Supporting Information). Accordingly, we initiated comprehensive cytological assays to investigate the role of COX7B in regulating cellular behavior and characteristics. DLD‐1 cells were selected for siRNA infection, and the efficiency was verified by using qPCR (Figure 4D). Afterward, flow cytometry was utilized to assess the biological impact of COX7B knockdown, which showed an increase in apoptotic cells and the percentage of cells in the G0/G1 phase, as well as a decrease in the S phase (Figure 4E,F). Cell proliferation experiments, including CCK‐8 and colony formation assays, indicated that COX7B promoted cell proliferation (Figure 4G,H). In addition, Trans‐well and cell wound scratch assays also demonstrated that COX7B promoted the invasion and migration of colorectal cells (Figure 4I; Figure S2F, Supporting Information). In summary, these findings suggest that COX7B may act as an oncogenic factor in the tumorigenesis of CRC.

### 2.4 scRNA‐seq Analysis Revealing the Tumor Microenvironment Concerning the MALMPS

**Figure 5:**
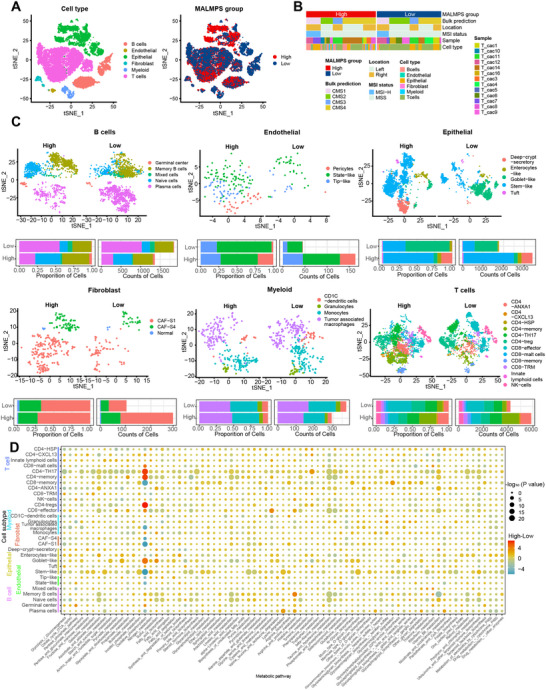
Single‑cell characterization of tumor microenvironments and metabolic activity between MALMPS subgroup. A) t‐SNE of CRC samples colored by six cell types (B cells, endothelial, epithelial, fibroblast, myeloid and T cells) and MALMPS group. B) Bar plot showing the clinical and molecular characteristics of stage II/III CRC sample in different MALMPS subgroup. C) t‐SNE of B cells, endothelial, epithelial, fibroblast, myeloid, T cells colored by cell subtypes and bar plots showing the proportions and counts of the six cell types. D) Heatmap showing the median of the differential score of metabolic pathways calculated by high versus low‐risk MALMPS in each cell subtypes. The size of the circle indicates ‐log_10_ (*P* value) and the color indicated the differential enrichment score in KEGG metabolic pathway.

Recent advances in single‐cell RNA‐seq (scRNA‐seq) have provided an avenue to explore the tumor microenvironment and metabolic reprogramming at a cellular resolution.^[^ ^15^, ^16^ ^]^ Here, we utilized the integrated scRNA‐seq and bulk RNA‐seq data from 13 stage II/III CRC patients to portray the cellular heterogeneity and metabolic remodeling among different MALMPS risk subgroups. Graph‐based clustering of merged and normalized cells identified robust, discrete clusters of epithelial cells, fibroblasts, endothelial cells, T cells, B cells, and myeloid cells based on canonical marker genes in CRC (**Figure** **5**A). Totally, we observed a significant clustering of epithelial cells in the high‐risk group, with a notably higher number of cell cycles in the G1 and G2M phases compared to the low‐risk group (Figure S3A, Supporting Information). The clinical and molecular annotations of the 13 patients at the single‐cell level are shown in Figure 5B. Specifically, the high‐risk group had an elevated count of endothelial cells (mainly state‐like cells), epithelial cells (mainly stem‐like cells), fibroblast cells (mainly CAF‐S1 subtype) and CD4^+^T cells (mainly CD4‐AXNA1, CD4‐memory), whereas the low‐risk group had a heightened number of B cells (mainly plasma cells), myeloid cells and CD8 ^+^T (mainly CD8 effector cells) (Figure 5C). Furthermore, we observed high expression of COX7B in epithelial malignant cells that clustered within the high‐risk group, providing further evidence of the malignant behavior of COX7B (Figure S3B, Supporting Information).

To understand the metabolic landscape of CRC with MALMPS, we evaluated the metabolic activity score of KEGG metabolic pathways at single cell level. Next, we computed the median of the differential metabolic score of each cell subtype (fibroblast‐normal and endothelial‐pericytes were excluded as cell counts <10) between high and low MALMPS risk groups (Figure 5D). The metabolic activity scores of cellular subtypes in the oxidative phosphorylation pathway exhibited significant variation between the high‐risk and low‐risk groups, showing an up‐regulation in epithelial and T cells, and down‐regulation in fibroblasts (Figure S3C, Supporting Information). Specifically, the oxidative phosphorylation activity in CD4‐Th17, CD4‐memory, CD4‐Treg, memory B cells, and goblet‐like epithelial cells was notably enriched in the high‐risk group. Moreover, taurine and hypotaurine metabolism were significantly reduced in CD4‐memory T cells, CD8‐effector T cells, memory B cells, and naïve B cells of the high‐risk subgroup, suggesting that impaired taurine metabolism in immune cells suppresses the antitumor activity within the tumor microenvironment (Figure S3D–F, Supporting Information).

### 2.5 Deciphering of Genetic Alteration Profiles with the MALMPS

The mutational landscape analysis revealed that the tumor driver genes related to the TGF‐β (Transforming Growth Factor‐beta) signaling pathway, including ARID1A, ACVR2A, and ACVR1B, exhibited a higher mutation frequency in the low MALMPS risk subgroups (*P* = 0.0122, 0.0093, and 0.0239, respectively, Figure S4A, Supporting Information). Intriguingly, mutation subtypes in these genes primarily consisted of loss of function mutations, including frameshift deletions/insertions, nonsense mutation, missense mutation, and splice‐site mutations (Figure S4B, Supporting Information), suggested the attenuated TGF‐β signaling in the low‐risk group. We further compared the tumor mutation burden (TMB) and somatic copy number alteration (SCNA) levels of stage II/III CRC and found that the normalized SCNA level in the high‐risk group was significantly higher than that in the low‐risk group (Figure S4C, Supporting Information). Subsequently, we extracted three mutational signatures from the genomic data with varying mutational activities and annotated them against the COSMIC‐V3 nomenclature by cosine similarity analysis (Figure S4D–F, Supporting Information). The extracted mutational signatures included polymerase epsilon exonuclease domain mutations (COSMIC‐10), spontaneous or enzymatic deamination of 5‐methylcytosine (COSMIC‐1), DNA mismatch repair deficiency‐related signatures (COSMIC‐6) (Figure S4G, Supporting Information). Mutational activities attributed to the COSMIC‐6 signature have a significant ascendance in the high MALMPS subgroup (*P* < 0.05, Figure S4H,I, Supporting Information).

### 2.6 Potential Biological Pathway Associated with the MALMPS

To elucidate the potential biological mechanisms of MALMPS, we identified the top positively and negatively enriched pathways associated with the MALMPS score in the GO, KEGG, and REACTOME datasets, using the normalized enrichment score (NES) from GSEA based on RNA‐seq data. The extracellular matrix and some organelle activities related to metabolism (e.g., mitochondria and ribosome) were found to be positively and negatively correlated with the MALMPS score, respectively, against the GO reference datasets. Similarly, the KEGG and REACTOME gene sets showed enrichment of processes related to the extracellular matrix, ribosomes, and mitochondria (Figure S5A, Supporting Information). Several oncogenic signaling pathways, such as the MAPK, JAK, and TGF‐β signaling pathways, were upregulated in the high‐risk group, whereas cancer suppression signaling pathways, such as the P53 pathway and DNA repair, were enhanced in the low‐risk group (Figure S5B, Supporting Information).

In the realm of proteomics analysis, kinases related to Wnt/β‐catenin signaling (GSK3B and PKD1) and MAPK signaling (PBK and MAPK15) were enriched in the high‐risk group, while TCA cycle metabolism (PDK2/3/4) and cell‐cycle signaling (AURKA, CDK1/2) were enriched in the low‐risk group (Figure S6A, Supporting Information). Certain phosphorylation pathways, such as the IGF1 and MAPK signaling pathways, were found to be enriched in the high‐risk group, while mTOR/MTOR was observed to be enriched in the low‐risk group (Figure S6B, Supporting Information). IGF‐1 can promote the growth and survival of cancer cells, as well as stimulate the formation of new blood vessels to support tumor growth. The mTOR is involved in the regulation of autophagy, a cellular process that degrades and recycles damaged or unnecessary cellular components. Our exploration successfully identified 46 distinct proteins showing differential expression, with 21 proteins (PCSK1, PLIN, CCL18, LDHAL6B, MAP3K8, and SERPINB5 et al.) were found to be up‐regulated and 25 (ITLN1, S1, TKTL2, and CASP5 et al.) down‐regulated when comparing the high‐risk and low‐risk groups (Figure S6C, Supporting Information).

### 2.7 MALMPS Model Prediction in Drug Sensitivity

**Figure 6:**
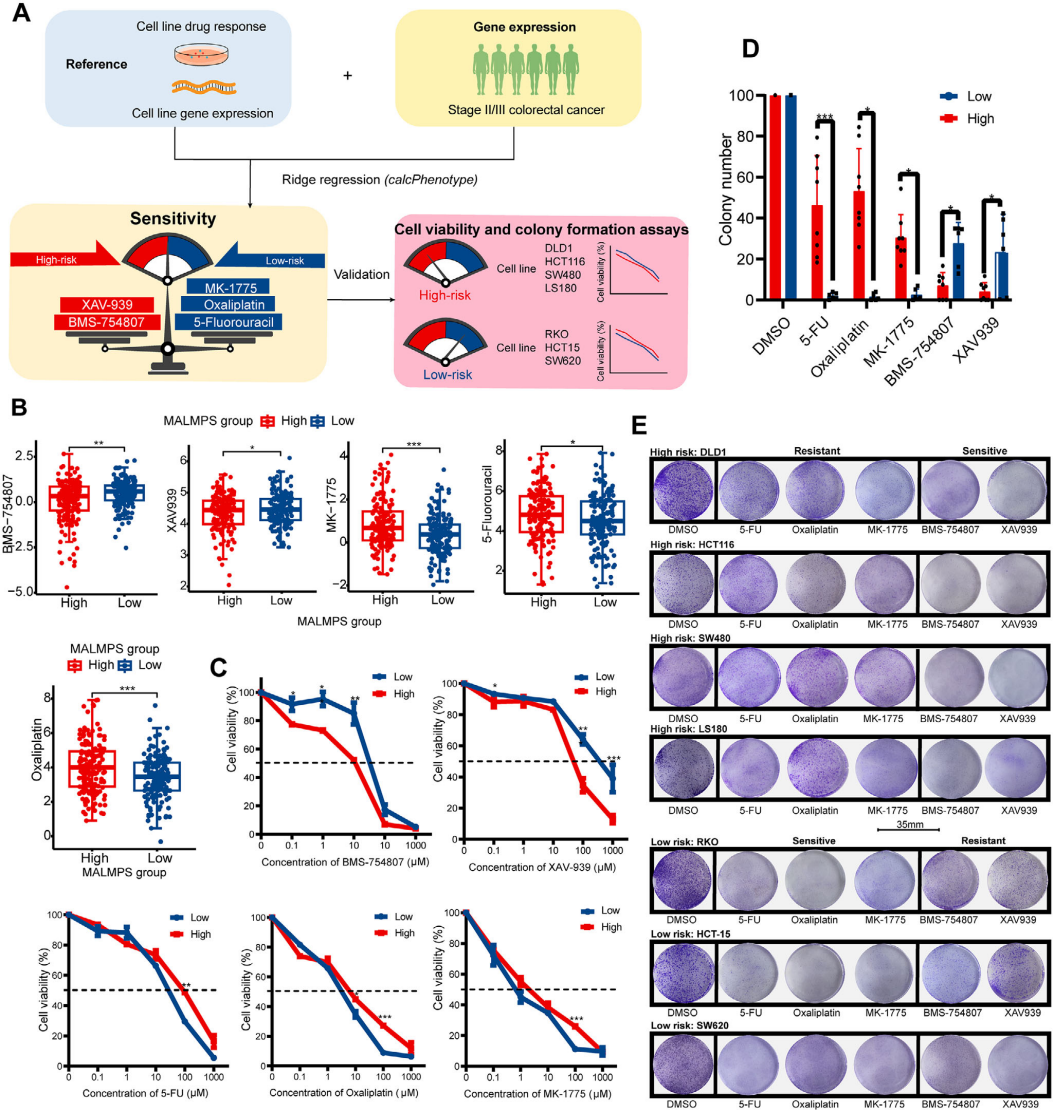
Drug sensitivity prediction and validation of MALMPS. A) Workflow of drug sensitivity prediction and validation. B) Relative distribution of drug sensitivity in fluorouracil, oxaliplatin, MK‐1775, BMS‐754807 and XAV939. C) Line charts showing cell viability in high‐ versus low‐risk groups with the identified five drugs and control drug. D) Bar plot showing the colony number in high‐ versus low‐risk groups with the identified five drugs and control drug. E) Representative staining images of colony number between risk groups among different cell lines. * *P* < 0.05, ** *P* < 0.01, *** *P* < 0.001.

Drug sensitivity analysis against GDSC2‐derived antineoplastic drugs was performed in different MALMPS risk groups with the ‘oncoPredict’ package (**Figure** **6**A). Patients with low risk were found to exhibit greater sensitivity to primary anticancer drugs commonly used in CRC therapy, such as 5‐fluorouracil and oxaliplatin. Additionally, cell cycle‐targeting drugs like MK‐1775 also demonstrated increased sensitivity in the low‐risk group, aligning with the findings from the GSEA results. Conversely, drugs such as BMS‐754807 (IGF‐1R inhibitors) and XAV939 (Wnt/β‐catenin inhibitors) were found to be more sensitive in the high‐risk group (Figure 6B). Our proteomic analysis also revealed dysregulation of IGF1 and Wnt/β‐catenin signaling in the high‐risk group (Figure S6A,B, Supporting Information). To further validate these findings, we selected seven CRC cell lines to perform drug sensitivity assays based on the risk grouping of MALMPS (high‐risk group: DLD1, HCT116, SW480, LS180; low‐risk group: RKO, HCT‐15, SW620). Cell lines from the high‐risk group displayed significantly lower colony numbers and reduced cell viability with treatment with XAV‐939 and BMS‐754807 than low‐risk group cell lines, while 5‐fluorouracil, oxaliplatin, and MK‐1775 showed the opposite effect (Figure 6C–E).

### 2.8 Metabolic Biological Activities, Metabolites Alteration, and Proteomic Difference Between MALMPS Groups

**Figure 7:**
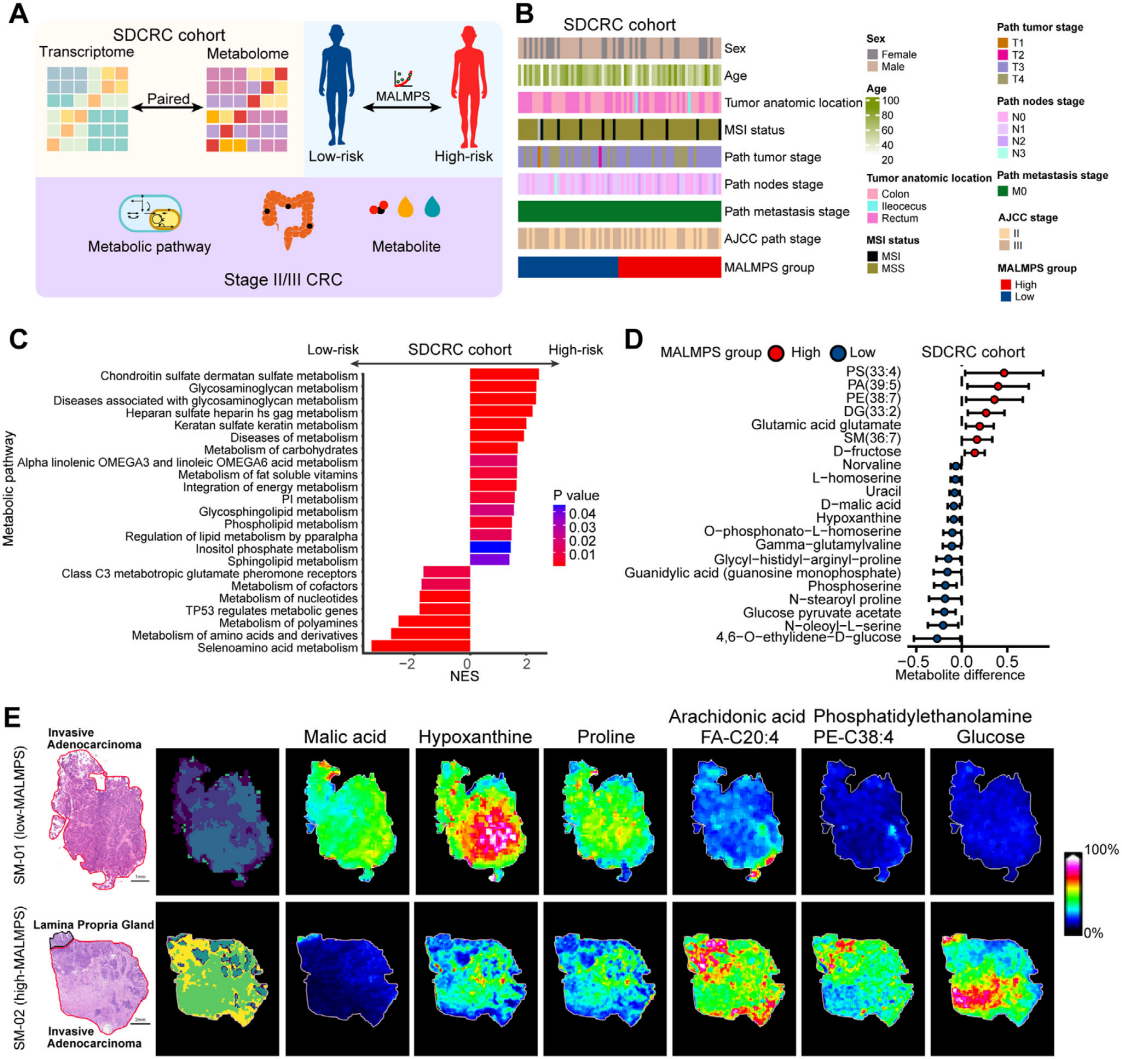
Metabolic characteristics of MALMPS. A) Scheme plot of metabolites profile in terms of MALMPS in SDCRC cohort. B) Clinical annotations of stage II/III colorectal cancer in SDCRC cohort. C) Metabolic pathway enrichment analysis between MALMPS risk groups against the REACTOME database. D) Forest plot illustrating metabolites alternation between risk groups in SDCRC cohort. E) Spatial metabolomic analysis further validated the metabolic reprogramming of tumor region in different MALMPS subgroups.

Metabolism biological activities, and metabolite profiles play important roles in understanding the metabolic reprogramming of CRC.^[^ ^17^, ^18^ ^]^ We utilized the paired metabolome and transcriptomics data of the 73 stage II/III CRC patients from our in‐house SDCRC cohort to identify the association between metabolite alteration and MALMPS risk groups (**Figure** **7A,B**; Table S4, Supporting Information). A total of 2633 metabolites (1244 positive and 1389 negative) with different molecule category was identified through the untargeted LC‐MS/MS approach (Figure S7A, Supporting Information). Initial correlation analyses between metabolite levels and the expression profiles of the MALMPS genes revealed dynamic bidirectional regulatory patterns between metabolic reprogramming‐associated genes (e.g., ARSA, SMPD1, COX7B) and key metabolites (Figure S7B, Supporting Information). Furthermore, GSEA analysis of the enriched REACTOME pathway among different MALMPS subgroups further supported findings from independent public datasets. We observed the enrichment of glycosaminoglycan (GAGs), carbohydrates, and a spectrum of lipid‐related pathways, notably encompassing phospholipid, fatty acids, and sphingolipid metabolism, were significantly pronounced in the high‐risk group. Concurrently, the metabolic pathways of nucleotides, cofactors, and amino acids emerged as enriched beacons within the low‐risk group (Figure 7C). In terms of metabolite abundance, phosphatidylethanolamine (PE), phosphatidylserine (PS), phosphatidic acid (PA), and sphingomyelin (SM), which were related to the lipid metabolism, also exhibited elevated relative levels in the high‐risk group. Metabolites involved in nucleotides (such as guanosine monophosphate, hypoxanthine, and uracil), and amino acids (such as proline, valine, and L‐serine) had an elevated score in the low‐risk group (Figure 7D).

Spatial metabolomics (SM) provides a visualization solution to explore the intratumor metabolic heterogeneity and cell metabolic interactions in the tumor microenvironment.^[^ ^19^, ^20^ ^]^ Here, we adopted the paired transcriptomic and spatial metabolomics to dissect the MALMPS subgroup‐related tumor metabolic dependency and heterogeneity. Two CRC samples were separately stratified into the low and high‐risk subgroup, and then subjected to MALDI‐MS‐based SM analysis (Method section, Table S5, Supporting Information). H&E images and spatial metabolites data‐driven PCA analysis, which was built based on region‐specific metabolite fingerprints, effectively stratified the two samples into distinct subgroups (Figure S7C, Supporting Information). Furthermore, we compared the metabolite level among the different MALMPS subgroups and found malic acid, hypoxanthine, and proline were enriched in the low‐risk group, whereas arachidonic acid (FA‐C20:4), phosphatidylethanolamine (PE‐C38:4) and glucose were enriched in the high‐risk group (Figure 7E). In summary, both metabolomics and spatial metabolomics analyses revealed distinct metabolic profiles across different MALMPS subgroups, highlighting the subgroup‐specific metabolic reprogramming pattern.

### 2.9 Development and Validation of the Prognostic Nomogram Model

**Figure 8:**
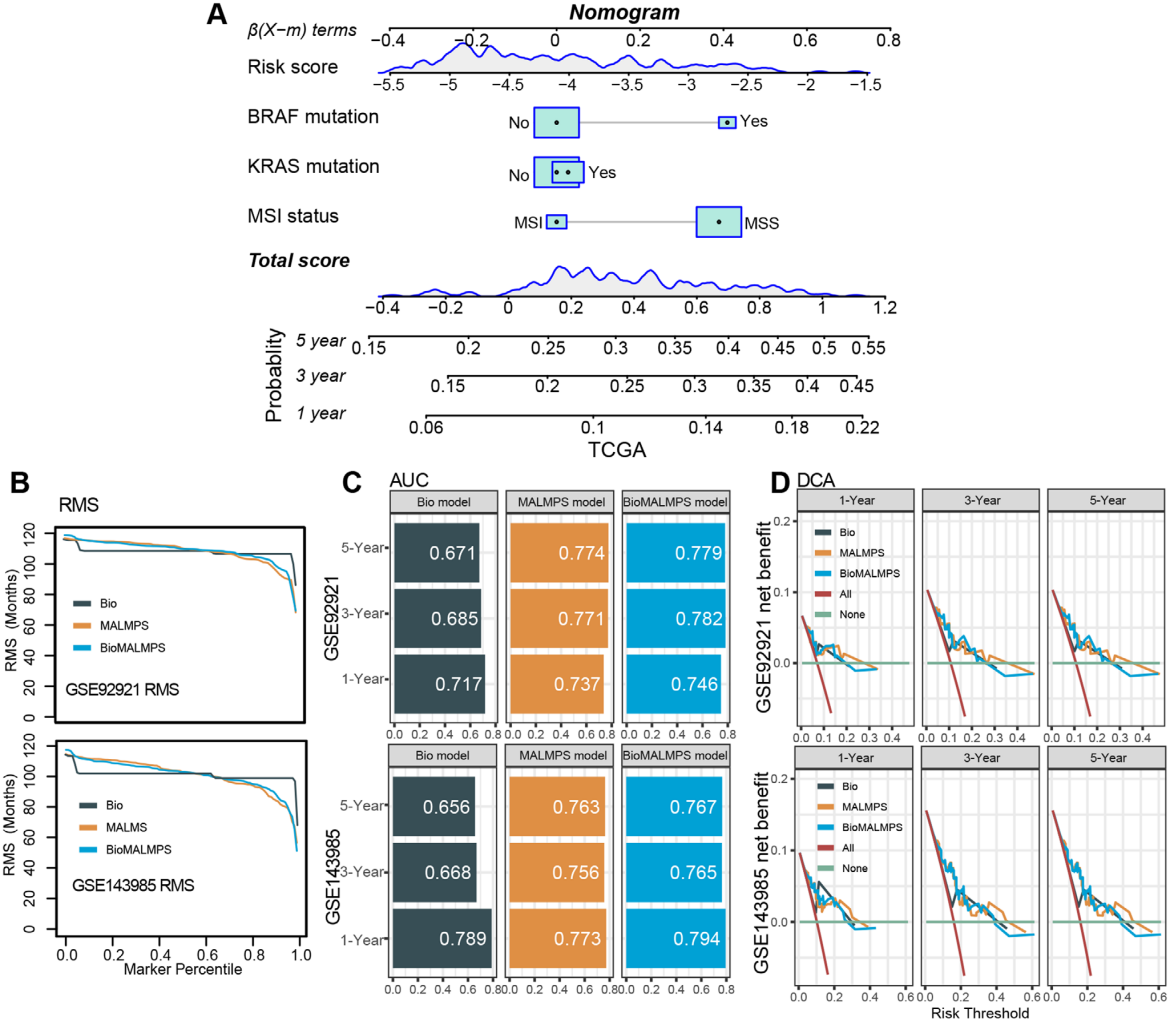
Combinations of MALMPS with molecular features. A) Prognostic nomogram predicting the probability of 1‐, 3‐, and 5‐year PFS in TCGA dataset. B) RMS plot of Bio, MALMPS and BioMALMPS in GSE92921 and GSE143985. C) AUCs of ROC curves of Bio, MALMPS and BioMALMPS for 1‐, 3‐, and 5‐year PFS probability. D) DCA of Bio, MALMPS and BioMALMPS model in terms of PFS for 1‐, 3‐, and 5‐year PFS prediction.

To provide a potential clinical tool for recurrence risk prediction within a certain period, we attempted to establish a nomogram model consisting of the MALMPS score and molecular features. We curated the common biomarkers recommended by the NCCN guidelines in the collected datasets, including MSI, BRAF, and KRAS mutations, to fit the Biomarker model (termed Bio) in the TCGA training set. These biomarkers were integrated with MALMPS to develop a nomogram model, referred to as BioMALMPS (**Figure** **8**A), which was subsequently validated using the GSE92921 and GSE143985 datasets. The continuous form of the MALMPS and BioMALMPS models showed a significantly improved estimation of PFS compared to the Bio model in the RMS analysis. The average slope of BioMALMPS was found to be larger than that of Bio and MALMPS (Figure 8B). Furthermore, we utilized ROC analysis to test the performance of the 1‐year, 3‐year, and 5‐year survival predictions derived by Bio, MALMPS, and BioMALMPS. There is a significant increase compared to Bio, with the highest value of BioMALMPS observed (Figure 8C). Net benefit was also apparent in the BioMALMPS model compared to the Bio and MALMPS models with a threshold less than 0.25 in DCA analysis (Figure 8D).

## 3 Discussion

This study developed the MALMPS model with 21 metabolic‐related prognostic genes through a combined approach using RSF and GBM, achieving the highest average C‐index in the independent validation datasets. Compared to traditional survival analysis methods, RSF has the advantages of handling high‐dimensional data and preventing overfitting.^[^ ^21^ ^]^ GBM combines the advantages of the Boosting algorithm and generalized regression models to gradually improve the predictive performance of the model.^[^ ^22^ ^]^ Among the genes in MALMPS, COX7B was identified as a relatively important gene in the MALMPS model, combined with the Chronos scores and prognostic analyses indicating its significance in tumor malignancy. Furthermore, an in vitro assay demonstrated the invasive and migratory effects of this gene on tumor cells. COX7B is a component of Complex IV in the mitochondrial respiratory chain, playing a crucial role in cellular respiration and energy production. As a part of the mitochondrial respiratory chain, it may promote cancer through its impact on mitochondrial function and metabolism, such as metabolic reprogramming and oxidative stress.^[^ ^23^ ^]^ Meanwhile, MALMPS had the highest C‐index level in our comparisons with common clinical and molecular features, and the AUCs of the 3‐year PFS were robust across all datasets. These findings demonstrated that our signature could be a promising biomarker for predicting the high‐risk recurrence population of stage II/III CRC in clinical practice.

Our findings showed that the low‐risk group had a higher frequency of inactivating mutation in genes belonging to TGF‐β signaling, suggesting reduced TGF‐β signaling activity in the low MALMPS subgroup. TGF‐β plays a critical role in tumor immune regulation by suppressing effector T cells, promoting regulatory T cells, and driving epithelial‐mesenchymal transition (EMT), which enhances tumor invasiveness and resistance to PD‐1 immunotherapy.^[^ ^24^ ^]^ Cao et al. demonstrated that taurine deficiency in the immune microenvironment resulted the T cell exhaustion and tumor progression,^[^ ^25^ ^]^ which aligns with our findings from scRNA‐derived metabolic landscape alteration in the high‐risk subgroup. On the other hand, the low‐risk group showed enrichment in cell cycle processes, and subsequent drug sensitivity analysis indicated their sensitivity to drugs targeting the cell cycle pathway. For example, 5‐fluorouracil (5‐FU) is an essential component of systemic chemotherapy for CRC, while XAV‐939 can disrupt Wnt signaling, affecting cellular proliferation and survival.^[^ ^26^, ^27^ ^]^

Our metabolic pathway enrichment revealed that sphingolipid (including glycosphingolipid and phospholipid) metabolism was enriched in the high‐risk group, with the abundance of PE, PS, PA, and SM acquired by LC‐MS. GAGs affect cancer progression by modulating growth factor signaling, extracellular matrix properties, and cancer cell spread, presenting opportunities for therapeutic targeting and drug delivery to enhance antitumor responses.^[^ ^28^, ^29^ ^]^ Phospholipids are essential components of cell membranes, which are involved in cellular signaling and can influence tumor growth and metastasis.^[^ ^30^, ^31^ ^]^ Cancer cells robustly incorporate exogenous fatty acids, which are subsequently remodeled into various lipid species, including phospholipids such as PA, PE, lysophosphatidylethanolamines (LPE), PI, and PS. Neutral lipids like triacylglycerols (TAG) and diacylglycerols (DAG) are formed, along with sphingolipids such as SM.^[^ ^32^ ^]^ Recent findings indicate that sphingolipid signaling can control intracellular complement activation, leading to inflammasome‐mediated metastasis, presenting a promising strategy for cancer therapy.^[^ ^33^, ^34^ ^]^ We found that glutamate is specifically enriched in the high‐risk group, which may suggest its significant role in the process of carcinogenesis and metastasis. Proliferating cancer cells predominantly depend on glutamine for their survival and proliferation.^[^ ^35^ ^]^ Glutamine functions as a crucial carbon source for the biosynthesis of lipids and metabolites through the tricarboxylic acid (TCA) cycle, while also providing nitrogen for the synthesis of amino acids and nucleotides.^[^ ^36^ ^]^ The observed negative correlation between L‐Homoserine, Phosphoserine, L‐Glutamate, and metabolic‐epigenetic regulatory gene clusters (e.g., ARSA, SMPD1, LHPP) likely stems from bidirectional feedback mechanisms at the metabolite‐enzyme interface. Metabolites negatively regulate the activity of epigenetic enzymes by disrupting cofactor availability (e.g., SAM, α‐KG) or through substrate competition, triggering cellular metabolic reprogramming to adapt to microenvironmental stress.^[^ ^37^ ^]^ This ultimately establishes a dynamic equilibrium between metabolic homeostasis and epigenetic stability.

Some limitations must be underscored with the current study, even though the results of our investigation were profound. First, although a total of 1442 patients were included with both microarray and RNA‐seq platforms, the follow‐up cohorts have reached conclusions with outcomes that have already materialized and new cases of our in‐house SDCRC cohort did not have adequate follow‐up outcomes, offering a foundation for future exploration. Second, several metabolic‐related genes from MALMPS in stage II/III CRC, and the linkage of MALMPS and other biological mechanisms, remain to be elucidated. Third, using the combination of metabolites and mRNAs might generate a more robust signature. Last, the effectiveness of the nomogram can be re‐validated in the future using our prospective cohort.

Collectively, our analysis established a stable and powerful signature based on machine learning algorithms by metabolic‐related genes. The findings indicate that MALMPS might be a valuable instrument for predicting the recurrence risk of stage II/III colorectal cancer within a specific timeframe, particularly for identifying individuals at high risk.

## 4 Experimental Section

### Study Design and Public Data Preprocessing for Prognostic Signature Establishment

The gene expression data and corresponding clinical features of CRC samples were collected from publicly available datasets of the NCBI GEO database (https://www.ncbi.nlm.nih.gov/geo/) and TCGA (https://cancergenome.nih.gov/). Transcriptomic and drug stativity data of CRC cell lines were downloaded from the Depmap database^[^ ^38^ ^]^ (https://depmap.org/portal/download/). The proteomic data of colon cancer samples were curated from the CPTAC dataset^[^ ^39^ ^]^ (https://www.cbioportal.org/study/summary?id = coad_cptac_2019).

A total of 1442 stage II/III CRC samples with RNA‐seq or microarray test, distributed in nine independent datasets, were used to establish the prognostic signature. A large retrospective follow‐up dataset from the Guinney et al. study,^[^ ^40^ ^]^ which includes GSE14333, GSE37892, GSE39582, and TCGA‐CRC, served as the training dataset for developing a recurrence prognostic prediction model. Independent validation datasets (GSE17536, GSE29621, GSE92921, GSE143985, GSE161158) from different nations were employed to select the optimal model combination and evaluate its predictive performance.

In this analysis, the data by excluding samples with zero survival time was refined, individuals who received preoperative chemotherapy and/or radiotherapy, and those with missing recurrence prognosis, staging, or transcriptome data. Only individuals with stage II/III colorectal cancer were retained, and survival time was standardized in months to ensure dataset homogeneity. A total of 1,000 samples were included in the training set to develop the prognostic model. Furthermore, 111, 40, 141, 59, and 91 samples were included in the five independent test cohorts to evaluate the effectiveness of the model. The processed data of the combined training set and five independent validation datasets are provided in the supplementary materials and Table S4 (Supporting Information).

### Collections and Preparation of SDCRC Cohort Clinical Specimen

73 stage II/III CRC samples from the in‐house Shandong colorectal cancer (SDCRC) cohort were collected and subjected to transcriptomics and metabolomics analysis. Another two CRC samples were utilized for paired transcriptomic and spatial metabolomics analysis. This cohort comprised patients who underwent surgical resection at the Shandong Hospital between August 2021 and June 2024. Post‐surgery, tissue samples were promptly snap‐frozen in liquid nitrogen within 30 min and subsequently stored at −80 °C until further analysis. The inclusion of these tissue samples in the study was sanctioned following the approval from the hospital ethics committee (Approval No. 2021‐176). Before their participation, each patient provided written informed consent. Importantly, all patients in this cohort were newly diagnosed with CRC and had not undergone any prior treatments for the disease. Comprehensive clinical information for each patient, such as age, sex, tumor location, tumor‐node metastasis, and AJCC staging, as well as the constructed MALMPS risk scores and respective groupings, are cataloged in Table S4 (Supporting Information). The transcriptomic data in TPM format was also log‐2 transformed and normalized into a Z score for further analysis.

The detailed methodologies for sample collection and preparation, along with the processes involved in transcriptome sequencing, LC‐MS/MS metabolomic, spatial metabolomic analysis, and subsequent data processing specific to the SDCRC cohort, are thoroughly documented in the supplementary methods.

### Metabolic‐Related Gene Signature Establishment

In this study, the outcome variables are survival information about recurrence, specifically focusing on the duration from surgery to either recurrence or follow‐up, along with the status of recurrence. The predictor variables in the signature were the expression level of the metabolic genes that meticulously screened. Since low‐expressed or nonvarying genes usually represent noise, the transcriptome data in the training dataset consisted of ≈6000 genes with the largest median absolute deviation (MAD). These genes were measured by at least one probeset in all datasets, and each gene was represented by the probe‐set with the largest MAD. 84 and 82 metabolic‐related pathways were extracted in the KEGG and REACTOME databases with 1715 and 2679 genes, respectively (Table S6, Supporting Information). A total of 1053 overlapping genes were utilized for further analysis. The intersection of the genes and performed univariate Cox regression analysis was performed with these genes. A total of 149 metabolic‐related prognostic genes were enrolled in the machine learning framework.

Ten separate machine learning algorithms and their combinations composed the machine learning framework. The ten algorithms included random survival forest (RSF), elastic network (Enet), Lasso, Ridge, stepwise Cox, CoxBoost, partial least squares regression for Cox (plsRcox), supervised principal components (SuperPC), generalized boosted regression modeling (GBM), and survival support vector machine (survival‐SVM). Six algorithms, including RSF, Enet, Lasso, Ridge, Stepwise Cox, and CoxBoost, could function with feature selection. A total of 83 combined algorithms based on 10‐fold cross‐validation (CV) or bootstrap resampling methods were utilized to select the optimal performance model. The implementation of the machine learning algorithm framework and corresponding hyperparameter optimization are shown in Table S7 (Supporting Information). The algorithm with the highest average C‐index across all validation datasets was regarded as the optimal model to generate the signature.

### Construction and Validation of the Nomogram

A novel nomogram for recurrence prediction of stage II/III CRC patients was constructed by the ‘rms’ package. The 2021 National Comprehensive Cancer Network guidelines recommend testing 7 biomarkers (ie, KRAS, NRAS, BRAF, microsatellite instability [MSI], mismatch repair [MMR], ERBB2 amplification, and NTRK fusion) to determine the optimal clinical management.^[^ ^41^ ^]^ There are three biomarkers present in both TCGA and validation datasets (GSE92921 and GSE143985). Through Cox proportional hazards regression analysis, these molecular features were systematically integrated with the established signature to develop a comprehensive prognostic model, which was subsequently visualized as a clinically applicable nomogram. The decision curve analysis (DCA) results could be performed to obtain the clinical net benefit of different models and all and no strategies.^[^ ^42^ ^]^ The prognostic performance of the comprehensive model was also compared in terms of the AUC and revealed by the restricted mean survival (RMS) curve.

### Statistical Analysis

The data processing, statistical analysis, and plotting were generated in R 4.2.2 software. Correlations between two continuous variables were evaluated via Spearman correlation coefficients. The Wilcoxon rank‐sum test or T test was applied to compare the difference between two groups for quantitative data. Analysis of variance or the Kruskal‐Wallis test was used to compare differences among four groups. Two‐sided Fisher exact tests were used to analyze categorical variables. The Cox proportional hazards model and Kaplan‐Meier analysis were performed with the ‘survival’ package. The receiver operating characteristic (ROC) curve was used to assess the prognosis classification performance of MALMPS. The area under the curve (AUC) was calculated via the ‘timeROC’ package. The comparisons between clinical and molecular traits and risk scores were implemented by the ‘compareC’ package. All statistical tests were two‐sided. *P *< 0.05 was considered statistically significant. The length of error bars represents 95% confidence intervals. Other relevant bioinformatics analyses and in vitro assays are detailed in the supplementary methods and Table S8 (Supporting Information).

### Availability of Data and Materials

The datasets supporting the conclusions of this article are available in TCGA (https://cancergenome.nih.gov/; https://www.cbioportal.org/), DepMap (https://depmap.org/portal/), GEO (https://www.ncbi.nlm.nih.gov/geo/), and CPTAC (https://cptac‐data‐portal.georgetown.edu/cptac) database. The raw transcriptomic data of the 73 CRC samples from SDCRC dataset have been deposited in the Genome Sequence Archive (https://ngdc.cncb.ac.cn/gsa/) under accession number HRA006800. Raw mass spectrometry data of metabolome of CRC samples in SDCRC have been deposited in the OMIX database of the National Genomics Data Center under accession number OMIX005924. The remaining data are available within the supplementary materials and reasonable request to corresponding author.

### Code Availability

Essential scripts for implementing machine learning‐based identification and validation procedure in multiple independent datasets are available on the Github website (https://github.com/chenhao‐qilu/machine‐learning).

## Conflict of Interest

The authors declare no conflict of interest.

## Author Contributions

H.C., Z.W., C.S. contributed equally to this work. W.C., M.L., F.F.T., and L.P.L. have conceived and designed the study. H.C., C.L.S., and Z.W. has contributed to the development of methodology. Z.W., Y.Z., Y.L., T.C.Z., Y.Z., Y.K.L., and Y.L. supported in acquisition, analysis and interpretation of data. Z.W., Y.L., M.L., F.F.T., W.C., L.P.L., and H.C. participated in writing, review, and/or revision of the manuscript. H.C., Z.W., F.F.T., and C.L.S. undertook the administrative, technical, or material support. All the authors read and approved the final version of the review.

## Supporting information



Supporting Information

Supporting Information

## Data Availability

The data that support the findings of this study are available in the supplementary material of this article.
