# Supplementary material for: MALMPS: A Machine Learning‐Based Metabolic Gene Prognostic Signature for Stratifying Clinical Outcomes and Molecular Heterogeneity in Stage II/III Colorectal Cancer
Source: Adv Sci (Weinh). 2025 Jul 17;12(37):e01333. doi: 10.1002/advs.202501333 (PMC12499476; doi:10.1002/advs.202501333)
Supplement: Supplementary file 1 — Supporting Information [file ADVS-12-e01333-s002.docx]

**Supplementary methods**

**Transcriptome data analysis**

The raw data of five validation GEO databases retrieved from the Affymetrix GPL570 platform were respectively processed by the robust multiarray averaging (RMA) algorithm with the ‘affy’ package. This function performs background correction, quantization, and normalization to ensure comparability across different microarray chips. Then ‘hgu133plus2.db’ package was used to annotate genes in the microarray data. For genes corresponding to multiple microarrays, the median value of these arrays is considered as the gene's expression level.

In SDCRC dataset, the raw paired end reads were trimmed and quality controlled by fastp with default parameters. Then clean reads were separately aligned to reference genome with orientation mode using HISAT2 software. The mapped reads of each sample were assembled by StringTie in a reference-based approach.

**Library preparation for transcriptome sequencing**

In SDCRC dataset, total RNA was extracted from the tissue using TRIzol® Reagent according the manufacturer’s instructions. Then RNA quality was determined by 5300 Bioanalyser (Agilent) and quantified using the ND-2000 (NanoDrop Technologies). Only high-quality RNA sample (OD260/280=1.8~2.2, OD260/230≥2.0, RIN≥6.5, 28S:18S≥1.0, >1μg) was used to construct sequencing library.

RNA purification, reverse transcription, library construction and sequencing were performed at Shanghai Majorbio Bio-pharm Biotechnology Co., Ltd. (Shanghai, China) according to the manufacturer’s instructions (Illumina, San Diego, CA). The RNA-seq transcriptome library was prepared following Illumina® Stranded mRNA Prep, Ligation from Illumina (San Diego, CA) using 1μg of total RNA. Shortly, messenger RNA was isolated according to polyA selection method by oligo(dT) beads and then fragmented by fragmentation buffer firstly. Secondly double-stranded cDNA was synthesized using a SuperScript double-stranded cDNA synthesis kit (Invitrogen, CA) with random hexamer primers (Illumina). Then the synthesized cDNA was subjected to end-repair, phosphorylation and ‘A’ base addition according to Illumina’s library construction protocol. Libraries were size selected for cDNA target fragments of 300 bp on 2% Low Range Ultra Agarose followed by PCR amplified using Phusion DNA polymerase (NEB) for 15 PCR cycles. After quantified by Qubit 4.0, paired-end RNA-seq sequencing library was sequenced with the NovaSeq Xplus sequencer (2 × 150bp read length).

**LC-MS/MS on metabolomic**

In SDCRC dataset, 50 mg solid sample was added to a 2 mL centrifuge tube and a 6 mm diameter grinding bead was added. 400 μLof extraction solution (methanol: water = 4:1 (v:v)) containing 0.02 mg/mLof internal standard (L-2-chlorophenylalanine) was used for metabolite extraction. Samples were ground by the Wonbio-96c (Shanghai wanbo biotechnology co., LTD) frozen tissue grinder for 6 min (-10°C, 50 Hz), followed by low-temperature ultrasonic extraction for 30 min (5°C, 40 kHz). The samples were left at -20°C for 30 min, centrifuged for 15 min (4°C, 13000 g), and the supernatant was transferred to the injection vial for LC-MS/MS analysis.

100 μL liquid sample was added to a 1.5 mL centrifuge tube with 400 μL solution (acetonitrile: methanol = 1:1(v:v)) containing 0.02 mg/mL internal standard (L-2-chlorophenylalanine) to extract metabolites.The samples were mixed by vortex for 30 s and low-temperature sonicated for 30 min (5°C, 40 KHz)。The samples were placed at -20°C for 30 min to precipitate the proteins.Then the samples were centrifuged for 15 min (4°C, 13000 g).The supernatant was removed and blown dry under nitrogen. The sample was then re-solubilized with 100 µL solution (acetonitrile: water = 1:1) and extracted by low-temperature ultrasonication for 5 min (5°C, 40 KHz), followed by centrifugation at 13000 g and 4°C for 10 min.The supernatant was transferred to sample vials for LC-MS/MS analysis.

The LC-MS/MS analysis of sample was conducted on a Thermo UHPLC-Q Exactive system equipped with anACQUITYHSS T3 column (100 mm × 2.1 mm i.d., 1.8 μm; Waters, USA) at Majorbio Bio-Pharm Technology Co. Ltd. (Shanghai, China).The mobile phases consisted of 0.1% formic acid in water:acetonitrile (95:5, v/v) (solventA) and 0.1% formic acid inacetonitrile: isopropanol:water (47.5:47.5, v/v)(solvent B). The flow rate was 0.40 mL/min and the column temperature was 40℃.

The UPLC system was coupled to a Thermo UHPLC-Q Exactive Mass Spectrometer equipped with an electrospray ionization (ESI) source operating in positive mode and negative mode.The optimal conditions were set as followed: source temperature at 400℃ ; sheath gas flow rate at 40 arb;Aux gas flow rate at 10 arb; ion-spray voltage floating (ISVF) at -2800V in negative mode and 3500V in positive mode, respectively; Normalized collision energy , 20-40-60V rolling for MS/MS. Full MS resolution was 70000, and MS/MS resolution was 17500. Data acquisition was performed with the Data DependentAcquisition (DDA) mode. The detection was carried out over a mass range of 70-1050 m/z.

**Quality control (QC) sample preparation and analysis‌**

In SDCRC dataset, QC samples were prepared by pooling equal volumes of all sample extracts. Each QC sample maintained the same volume as individual test samples and underwent identical processing and analytical procedures.

For instrumental analysis, QC samples were interspersed at regular intervals (every 5-15 test samples) to monitor system stability throughout the entire analytical run. This QC placement strategy allowed for continuous assessment of detection process reliability.

**Total ion chromatogram (TIC)**

The chromatographically separated components continuously enter the mass spectrometer, where sequential scanning performs data acquisition. Each scan generates a mass spectrum, and the summation of all ion intensities from individual spectra yields the total ion current intensity. The TIC is then plotted with retention time as the x-axis and cumulative ion intensity as the y-axis.

**Raw data processing and metabolite identification**

The raw data were imported into the metabolomics processing software Progenesis QI v3.0 (Waters Corporation, Milford, USA) for a series of preprocessing steps, including baseline filtering, peak detection, integration, retention time correction, and peak alignment. These steps culminated in the generation of a data matrix containing information such as retention times, mass-to-charge ratios (m/z), and peak intensities. Subsequently, the software was utilized to perform feature peak library searching and identification. The MS and MS/MS mass spectrometry information was matched against metabolic databases, with the MS mass error set to less than 10 ppm. Metabolites were identified based on the matching scores of the secondary mass spectrometry. The primary databases used for this purpose included mainstream public databases such as http://www.hmdb.ca/ and https://metlin.scripps.edu/.

In untargeted metabolomics studies, multiple internal standards (IS) are employed to assess the stability of the experimental workflow and ensure data quality. According to the Law of Large Numbers and Central Limit Theorem, data points falling outside the 95% confidence interval (i.e., exceeding ±1.96 standard deviations from the mean) are statistically defined as outliers. Consequently, the z-score is widely used to evaluate data stability and identify outliers.

To account for variability in mass spectrometric response intensities across different internal standards, the stability of the workflow is systematically assessed by calculating whether the z-score for each IS lies within ±2 standard deviations. This threshold (a stricter criterion than the theoretical 1.96) is applied to robustly monitor experimental consistency and mitigate batch effects.

**Metabolomic data processing**

The data were analyzed through the free online platform of majorbio choud platform (cloud.majorbio.com). Metabolic features detected at least 80% in any set of samples were retained. After filtering, minimum metabolite values were imputed for specific samples in which the metabolite levels fell below the lower limit of quantitation, and each Metabolic features were normalized by sum. To reduce the errors caused by sample preparation and instrument instability, the response intensity of the sample mass spectrum peaks was normalized by the sum normalization method, and then the normalized data matrix was obtained. Meanwhile, variables with relative standard deviation (RSD) > 30% of QC samples were removed, and log10 processing was performed to obtain the final data matrix for subsequent analysis.

**Spatial metabolomic analysis**

CRC tissues were first embedded in OCT compound and then cut into 14 μm tissue sections at -20℃ on a cryostat microtome (Thermo CryoStar NX50 NOVPD, Bre men, Germany). The frozen tissue sections were then naturally and quickly placed on ITO-coated conductive side of glass slide for subsequent MALDI-MS analysis. 3.0 mg/mL 1,5-DAN in ACN/H2O (80:20, v/v) was sprayed onto the tissue section at a flow rate of 100 μL/min. The nitrogen jet pressure and temperature were set at 0.6 MPa and 50 °C, the temperature and height of the nozzle were 15 mm/s and 20 mm, respectively. After 15 cycles of matrix solution spraying, MSI experiment was carried out on rapi-flex MALDI-TOF/TOF mass spectrometer (Bruker Daltonics, Billerica, MA) in positive and negative ions. The spatial resolution was set to 100 μm. Ion source voltage was set to 16 kV, laser frequency was set to 5000 Hz, sampling rate was set to 2 GS/s. SCiLS Lab 2018b software (GmbH, Bremen, Germany) was used to visualize the spatial distributions of metabolites.

**Signature characterization**

The RNA-sequencing data of TCGA-CRC patients used for signature characterization were curated from UCSC Xena (https://xenabrowser.net/datapages/), and transformed into TPM format and log-2 transformation. All gene expression was transformed into a normalized Z score among samples when training and using the models.

**Chronos score analysis in colorectal cell lines**

We utilized the Chronos scores to infer the gene fitness effects within cell lines following knockout of the signature gene (https://github.com/broadinstitute/chronos)[1]. The Chronos algorithm employs a model of cell proliferation dynamics after clustered regularly interspaced short palindromic repeats (CRISPR) gene knockout to infer gene knockout fitness effects. The score produced by the Chronos algorithm gauges the impact of a particular gene knockout on cell proliferation and fitness.

**Spatial transcriptomic analysis and cell-type estimation**

The processed spatial transcriptomic (ST) data was collected from Wu et al. study[2]. The ST data tissue structure annotations in the eight samples (paired colorectal cancer and liver metastasis) from two untreated patients and two neoadjuvant chemoradiotherapy patients were sourced from the author. We employed SCTransform for data normalization, RunPCA for dimension reduction, FindNeighbors and FindClusters for clustering the ST spots, and RunUMAP for data visualization. The subcell types in ST spots were annotated with ssGSEA algorithm.

**Cell culture and transfection**

The LS180 cell line (RRID: CVCL_0397) was procured from the Cell Resource Center at Peking Union Medical College, a part of the National Science and Technology Infrastructure, specifically the National Biomedical Cell-Line Resource (NSTI-BMCR). The cells were cultured in MEM (KeyGEN BioTECH). The HCT116 cell line (RRID: CVCL_0291), obtained from Procell, was grown in McCoy’s 5A medium (KeyGEN BioTECH). Additionally, other colorectal cell lines [RKO (RRID: CVCL_0504), HCT15 (RRID: CVCL_0292), SW480 (RRID: CVCL_0546), SW620 (RRID: CVCL_0547), DLD-1 (RRID: CVCL_0248)] were sourced from ATCC and were cultured in RPMI-1640 (Gibco) medium, except for RKO, which was cultured in DMEM (Gibco). All culture media mentioned above were supplemented with 10% fetal bovine serum (PAN), penicillin (100 U/mL, Thermo Fisher), and streptomycin (100 U/mL, Thermo Fisher). The cells were maintained in a 95% air and 5% CO2 environment at 37°C.

All human cell lines were authenticated using STR (or SNP) profiling within the last three years (Supplement Material), and the cells used in all experiments were mycoplasma-free cells.

**Real-time quantitative PCR**

Total RNA from cells was isolated with TRIzol reagent and reverse transcribed into cDNA by HiScripIII RT SuperMix following the manufacturer’s instructions. We used an Applied Biosystems QuantStudio 1 Real Time PCR system (Applied Biosystems, ThermoFish) to perform quantitative real-time polymerase chain reaction (qRT-PCR) with ChamQ Universal SYBR qPCR Master Mix. All reagents were purchased from Vazyme, China. The relative expression levels of mRNA were calculated by using the 2^−ΔΔCt^ method, and higher 2^−ΔΔCt^ reflects higher expression.

**Cell counting kit-8 (CCK-8) assays**

We seeded 3,000 cells per well into a 96-well plate and added 10 µL/well of CCK-8 reagent (DojinDo, Japan) after cell adhesion. After incubation at 37°C for 2 hours, the absorbance value of each well was measured at a wavelength of 450 nm selected by Multiskan Sky (Thermo Scientific) at 0 hours, 24 hours, 48 hours, 72 hours and 96 hours. The cell viability was calculated and then mapped with GraphPad Prism 8 software. All experiments were repeated three times.

**Transwell assay**

We added 600 μl complete 1640 culture medium to the lower compartment and 200 μl cell suspensions of serum-free medium (migration: 4×10^5^/mL, invasion: 12×10^6^/mL) to the top chamber of an 8-mm hole, with Matrigel for invasion assays or without Matrigel for migration assays. After incubation for 24 hours, the upper chambers were fixed in 4% paraformaldehyde for 30 minutes and stained with 0.1% crystal violet for 30 minutes. Light microscopy was used to count the cells in random fields.

**Apoptosis and cell cycle analysis**

Apoptosis and the cell cycle status were assessed using apoptosis (BD, Pharmingen™ PE Annexin V Apoptosis Detection Kit I) and cell cycle kits (KeyGEN BioTECH, Cell Cycle Detection Kit), respectively. Samples were prepared according to the manual and analyzed by a CytoFLEX S Flow Cytometer (Beckman Coulter).

**Cell wound scratch assay**

We inoculated cells in 6-well plates. When cells covered the bottom of the well, we used sterile 10 µl pipette tips to make three wounds per well. The same culture system (RPMI-1640 medium + 1% FBS) was used to continuously cultivate the cells for 72 hours. We recorded scratch width in three randomly chosen microscopic fields at specific time points (0 hours, 24 hours, 48 hours, 72 hours). The wound closure ratio was calculated as the ratio of the migration distance to the starting wound distance.

**scRNA‑seq identification of cell clusters and metabolic pathway enrichment**

Processed single cell RNA sequence (scRNA-seq) data were obtained from Khaliq et al. study[3]. Samples with stage II/III CRC were included in the analysis and obtained a total of 19695 cells after a series filter criterion according to the previous characterization. R package ‘Seurat’ offers dimensionality reduction capabilities through t-distributed stochastic neighbor embedding (t-SNE), allowing for the visualization of cell topology in high-dimensional space on a two-dimensional plane. This non-linear method helps us to identify distinct cell clusters and annotate different cell types between risk groups in visual representations[4]. Cell subtype cluster identification was based on the Khaliq et al. annotation. The ‘scMetabolism’ package quantifies metabolic activity at the single-cell level by utilizing KEGG or REACTOME database based on standard single-cell matrix file. It leverages the VISION algorithm to assess each cell and calculate a score representing the cell's activity within various metabolic pathways[2].

**Estimation and validation of drug sensitivities**

The ‘oncoPredict’ package was used to build the prediction procedure. The imputations were performed based on the expression matrix of a training set with known drug treatment information against the Genomics of Drug Sensitivity in Cancer (GDSC) database[5]. The drug sensitivity scores of the samples were calculated using ridge regression.

**Cell viability assay**

Cell lines were stratified into low- and high-risk group based on their corresponding risk scores, as determined by the MALMPS with transcriptomic data for drug sensitivity assays. The cells cultured with the corresponding medium, which contained 10% serum, were digested to form a single cell suspension and evenly inoculated into 24-well plates. The volume of the medium per well was 500 μL. After culturing for 24 hours, when all cells adhered to the cell wall, we changed the culture medium to the same volume with different drug concentrations (0, 0.1, 1, 10, 100, and 1000 μM) (Table S8). After continuous culture for 48 hours, 500 μL basic medium containing 50 μL CCK-8 solution was added to each well and then incubated at 37°C for 2 hours. The absorbance values of each pore were measured three times at a wavelength of 450 nm selected by an Infinite® M Plex multimode microplate reader and TECAN monochromator. The cell viability at different drug concentrations was calculated and then mapped with GraphPad Prism 8 software. All experiments were repeated three times.

**Colony formation assays**

For functional assays, we seeded 3000 cells in one well of 6-well plates and replaced the culture medium with fresh medium every 3 days until the 7th day.

For drug sensitivity assays, we inoculated 10,000 cells per well in 12-well plates and allowed them to adhere to the cell wall for 24 hours. Next, we replaced the medium with a drug at its IC50 concentration and incubated for another 48 hours.

After cultivation for the appropriate time, the 6-well plates and 12-well plates were washed twice with 4°C precooled PBS, fixed with 4°C precooled 4% paraformaldehyde for 30 minutes, stained with crystal violet for 30 minutes, and photographed after drying. We calculated the numbers of colonies with ImageJ software. Each experiment was performed in triplicate.

**Genomic operation on mutational signature**

The samples in training dataset were stratified into high-risk group and low-risk group according to the median MALMPS score. TCGA-CRC genomic data was curated from ‘TCGAbiolink’ package. Mutational landscape depiction and signatures extraction were both applied in the ‘maftools’ package. ExtractSignatures function based on Bayesian variant nonnegative matrix factorization factorized the mutation portrait matrix into two nonnegative matrices ‘signatures’ and‘contributions’, where ‘signatures’ represent mutational processes and ‘contributions’ represent the corresponding mutational activities[6]. The SignatureEnrichment function can automatically determine the optimal number of extracted mutational signatures and assign them to each sample based on the mutational activities. The extracted mutational portrait of CRC was compared and annotated by cosine similarity analysis against the Catalogue of Somatic Mutations in Cancer (COSMIC-V2) database[7]. The somatic mutation and copy number alteration (SCNA) segment data downloaded from Teresa et al. study[8].

**Gene set enrichment analysis**

The correlation coefficients between the MALMPS scores and each gene expression acquired were calculated. The sorted correlation coefficients were used as the ranked gene list input to perform gene set enrichment analysis (GSEA) via ‘clusterProfiler’ package against GO, KEGG and REACTOME reference gene set[9]. False discovery rate (FDR) < 0.05 was considered to be the cutoff of enriched gene sets.

The ‘limma’ package was used to evaluate differential expression of more than 18,000 genes in samples with different risk groups and immune infiltration patterns. The gene expression data were fed into lmFit and eBayes functions to calculate the differential statistics with the package[10]. The ranked logFC produced by limma was used to implement GSEA with fast GSEA algorithm against the curated 84 KEGG metabolic gene sets.

**Proteomics and phosphoproteomic analysis of different MALMPS groups**

CPTAC generates comprehensive proteomics and genomics data primarily from clinical cohorts. In our study, we sourced transcriptomic, proteomic, and clinical data from the cBioPortal platform (https://www.cbioportal.org/study/summary?id=coad_cptac_2019). Focusing on a subset of 79 patients diagnosed with stage II/III colorectal cancer, we employed the MALMPS to calculate risk scores. Subsequently, these patients were stratified into different risk groups using a consistent methodology. The proteomics data used for MALMPS gene identification was downloaded from ProteomeXchange Consortium (identifier: PXD046566).

The PTM-Signature Enrichment Analysis (PTM-SEA) is a modified version of ssGSEA designed for site-specific signature analysis by scoring PTMsigDB's bi-directional signature-sets. In the data matrix, each row represents a single phosphorylation site confidently localized to a specific amino acid residue, with measured abundances across samples specified in columns. KSEA (Kinase-Substrate Enrichment Analysis) is a method used to study the enrichment of protein kinase substrates. This method reveals the role of kinases in cellular signal transduction by analyzing the enrichment of protein kinase substrates, which was performed in ‘KSEAapp’ package. For the identification of differentially expressed proteins, we utilized the ‘limma’ package, setting stringent criteria with a p-value threshold of less than 0.05 and a fold change (FC) greater than 1.5.

**References**

1. Dempster JM, Boyle I, Vazquez F, Root DE, Boehm JS, Hahn WC, Tsherniak A, McFarland JM. Chronos: a cell population dynamics model of CRISPR experiments that improves inference of gene fitness effects**.** Genome Biol. 2021;22**:**343.

2. Wu Y, Yang S, Ma J, Chen Z, Song G, Rao D, Cheng Y, Huang S, Liu Y, Jiang S, et al. Spatiotemporal Immune Landscape of Colorectal Cancer Liver Metastasis at Single-Cell Level**.** Cancer Discov. 2022;12**:**134-153.

3. Khaliq AM, Erdogan C, Kurt Z, Turgut SS, Grunvald MW, Rand T, Khare S, Borgia JA, Hayden DM, Pappas SG, et al. Refining colorectal cancer classification and clinical stratification through a single-cell atlas**.** Genome Biol. 2022;23**:**113.

4. Butler A, Hoffman P, Smibert P, Papalexi E, Satija R. Integrating single-cell transcriptomic data across different conditions, technologies, and species**.** Nat Biotechnol. 2018;36**:**411-420.

5. Maeser D, Gruener RF, Huang RS. oncoPredict: an R package for predicting in vivo or cancer patient drug response and biomarkers from cell line screening data**.** Brief Bioinform. 2021;22.

6. Mayakonda A, Lin DC, Assenov Y, Plass C, Koeffler HP. Maftools: efficient and comprehensive analysis of somatic variants in cancer**.** Genome Res. 2018;28**:**1747-1756.

7. Kandoth C, McLellan MD, Vandin F, Ye K, Niu B, Lu C, Xie M, Zhang Q, McMichael JF, Wyczalkowski MA, et al. Mutational landscape and significance across 12 major cancer types**.** Nature. 2013;502**:**333-339.

8. Davoli T, Uno H, Wooten EC, Elledge SJ. Tumor aneuploidy correlates with markers of immune evasion and with reduced response to immunotherapy**.** Science. 2017;355.

9. Subramanian A, Tamayo P, Mootha VK, Mukherjee S, Ebert BL, Gillette MA, Paulovich A, Pomeroy SL, Golub TR, Lander ES, Mesirov JP. Gene set enrichment analysis: a knowledge-based approach for interpreting genome-wide expression profiles**.** Proc Natl Acad Sci U S A. 2005;102**:**15545-15550.

10. Ritchie ME, Phipson B, Wu D, Hu Y, Law CW, Shi W, Smyth GK. limma powers differential expression analyses for RNA-sequencing and microarray studies**.** Nucleic Acids Res. 2015;43**:**e47.

**Supplementary figures**


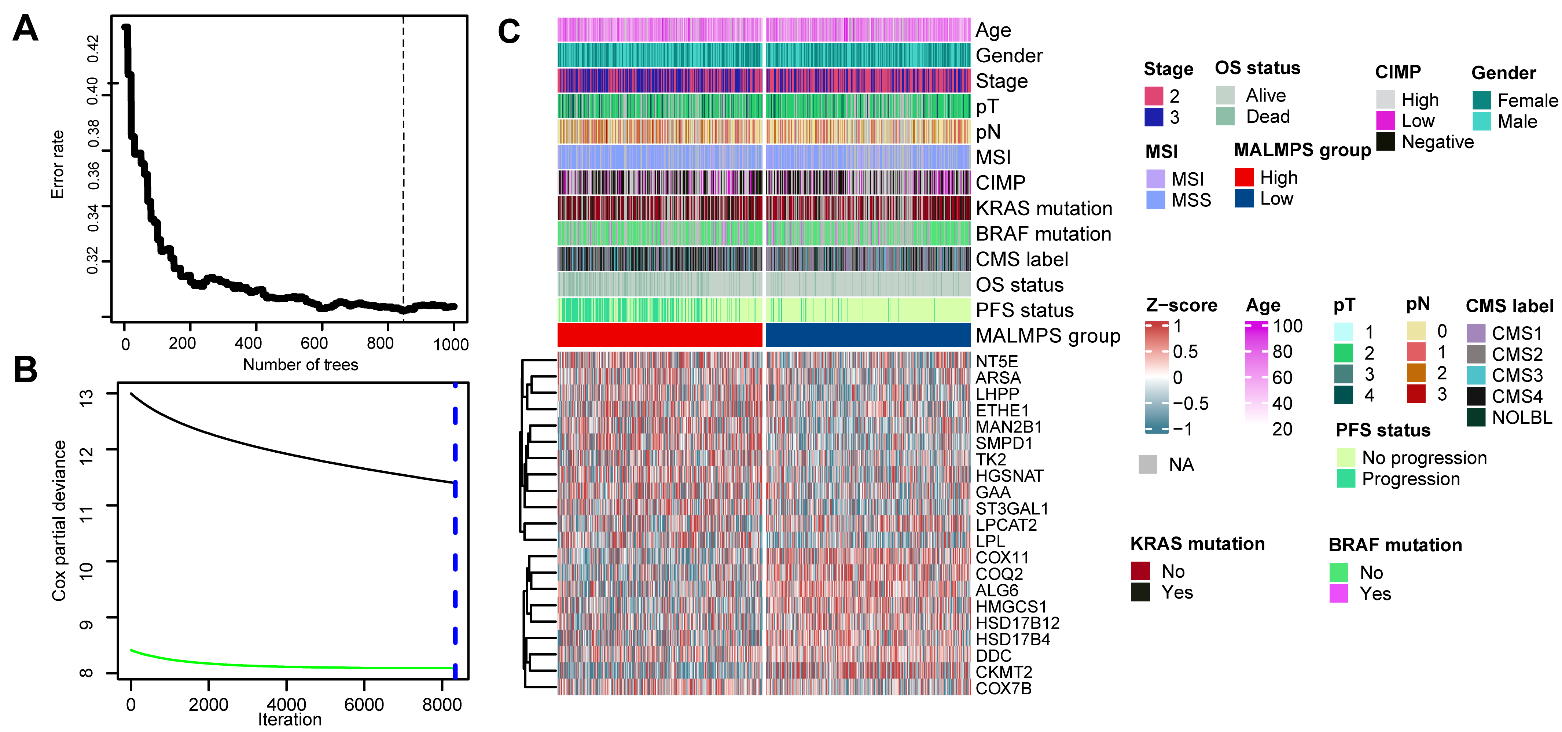


**Figure S1.** (A) The number of trees for generating the signature with minimal error via RSF algorithm. (B) Performance curves showing the optimal number of boosting iterations in GBM algorithm. (C) Heatmap revealing the differences of clinicopathological parameters in the high- and low-risk groups according to the expression patterns of the identified signature.


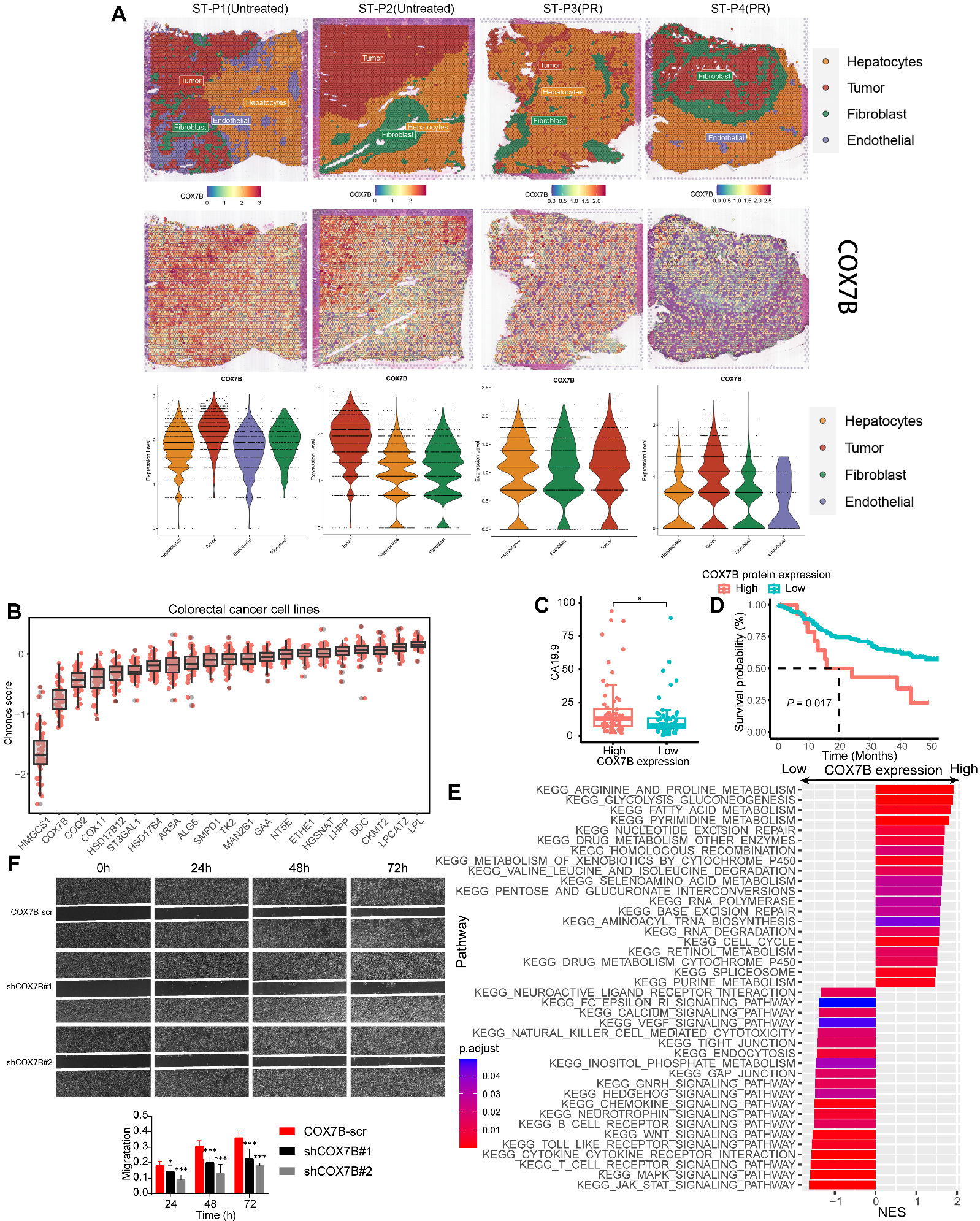


**Figure S2.** (A) Heatmap and violin plot showing the unsupervised clustering analysis distribution and COX7B expression of ST among different cell types in liver metastasis. (B) Boxplot showing Chronos scores of the 21 genes in MALMPS. (C) Distribution of CA199 levels in high- versus low-risk groups. (D) Kaplan-Meier survival curve of PFS between patients with high COX7B and low COX7B protein expression.(E) Top 40 KEGG pathway enrichment annotation between the high- and low-expression group of COX7B. (F) Representative staining images and barplot of scratch migration differences within COX7B-scr, shCOX7B#1 and shCOX7B#2 groups.


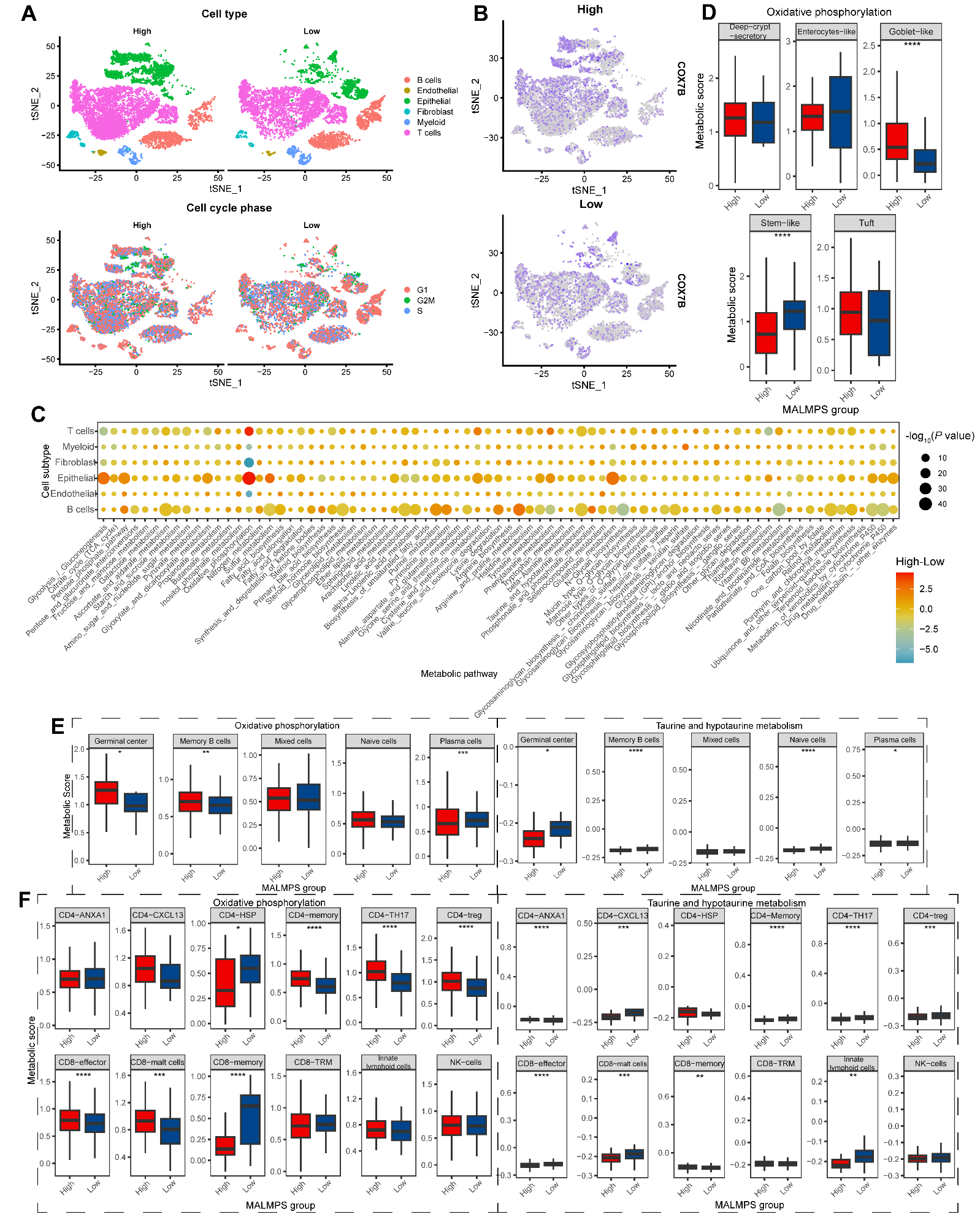


**Figure S3.** (A) t-SNE of CRC samples colored by six cell types (B cells, endothelial, epithelial, fibroblast, myeloid and T cells) and cell cycle phase (G1, G2M and S) between MALMPS groups. (B) t-SNE of CRC samples colored by COX7B expression level between MALMPS groups. (C) Heatmap showing the median of the differential score of metabolic pathways calculated by high versus low-risk MALMPS for the profile of each cell subtype. The size of the circle indicates -log10 (P value) and the color indicated the differential enrichment score in KEGG metabolic pathway. (D) Box plot illustrating the differences in oxidative phosphorylation metabolic scores between MALMPS groups in types of epithelial cells. (E) Box plot illustrating the differences in oxidative phosphorylation and taurine and hypotaurine metabolism scores between MALMPS groups in types of B cells. (F) Box plot illustrating the differences in oxidative phosphorylation and taurine and hypotaurine metabolism scores between MALMPS groups in types of T cells. * means *P* < 0.05, ** means *P* < 0.01, *** means *P* < 0.001, **** means *P* < 0.0001.

**
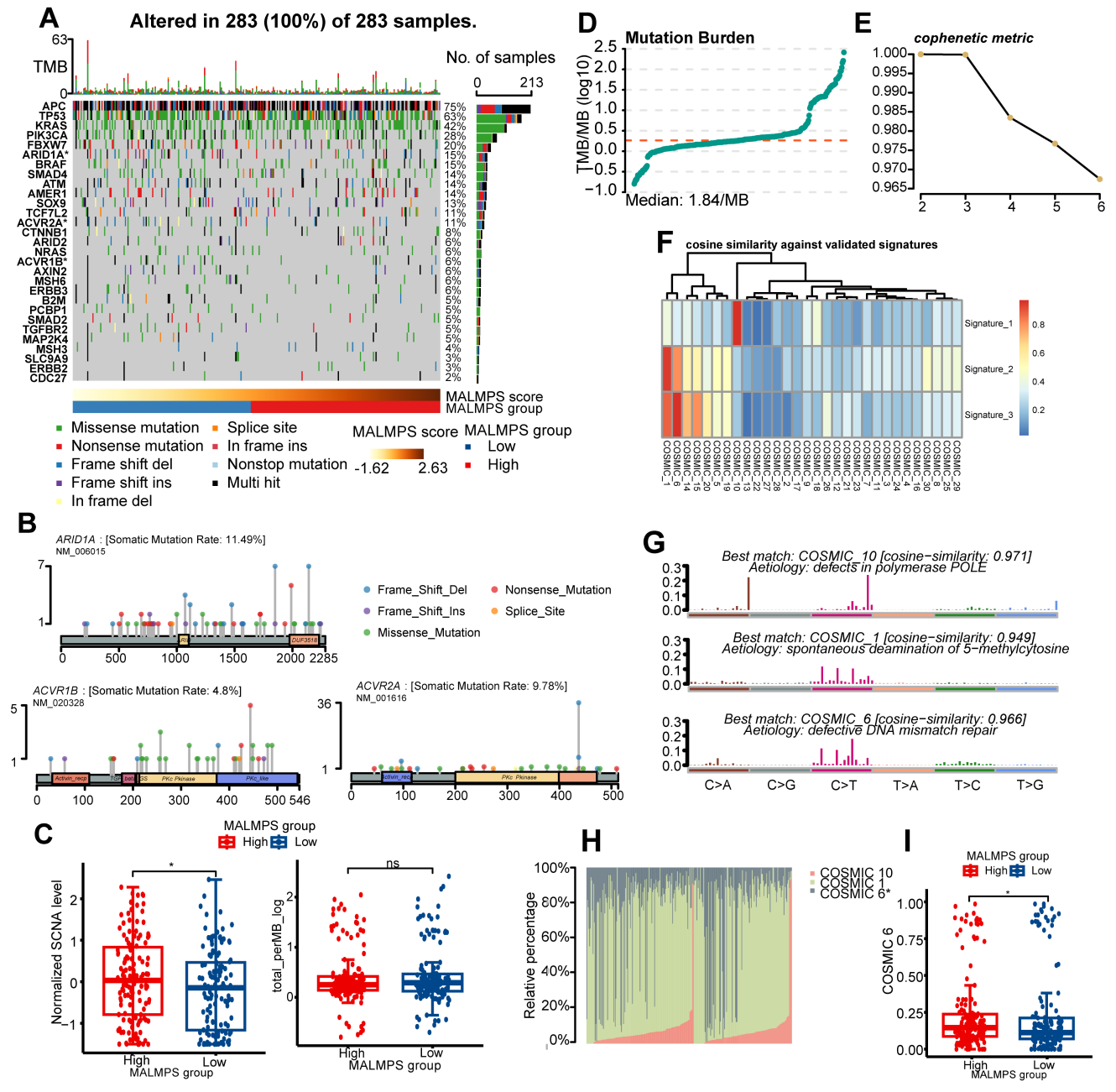
**

**Figure S4.** (A) Mutational landscape of MALMPS in TCGA-CRC stratified by low versus high MALMPS group. Individual patients were represented in each column, and they were sorted by MALMPS score from left to right. (B) Lollipop chart of mutated gene AR1D1A, ACVR1B and ACVR2A. (C) Normalized distribution of SCNA and TMB levels in high- versus low-risk groups. (D) TMB landscape sorted by value. (E) NMF cophenetic metric curve used for selection of matched mutational signatures. (F Heatmap showing the optimal matched signatures against COSMIC database signatures by cosine similarity. (G) The mutational activities of corresponding extracted mutational signatures (signature 10, 1, and 6, matched with COSMIC database). (H) Relative percentage barplot of extracted mutational signatures in low- versus high-risk group. (I) Relative distribution of mutational signature COMSIC 6 in high- versus low-risk groups. * means *P* < 0.05.


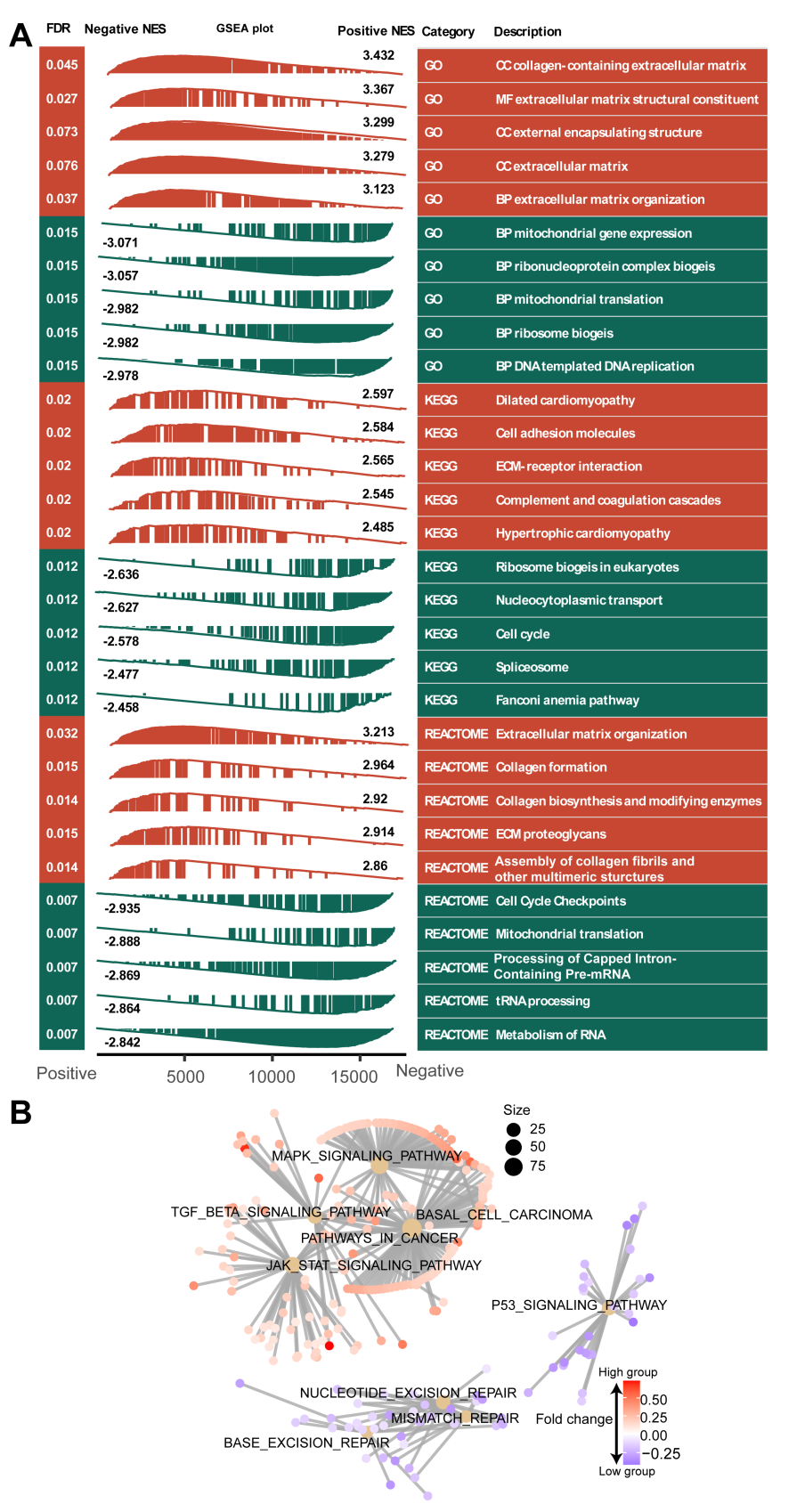


**Figure S5.** (A) Top 5 pathways that were positively and negatively correlated with MALMPS against GO, KEGG and REACTOME databases, respectively. (B) Interaction networks of hallmark extracted from KEGG database between risk groups. The color represented the direction of fold change. The size of circle represented the number of genes enriched.


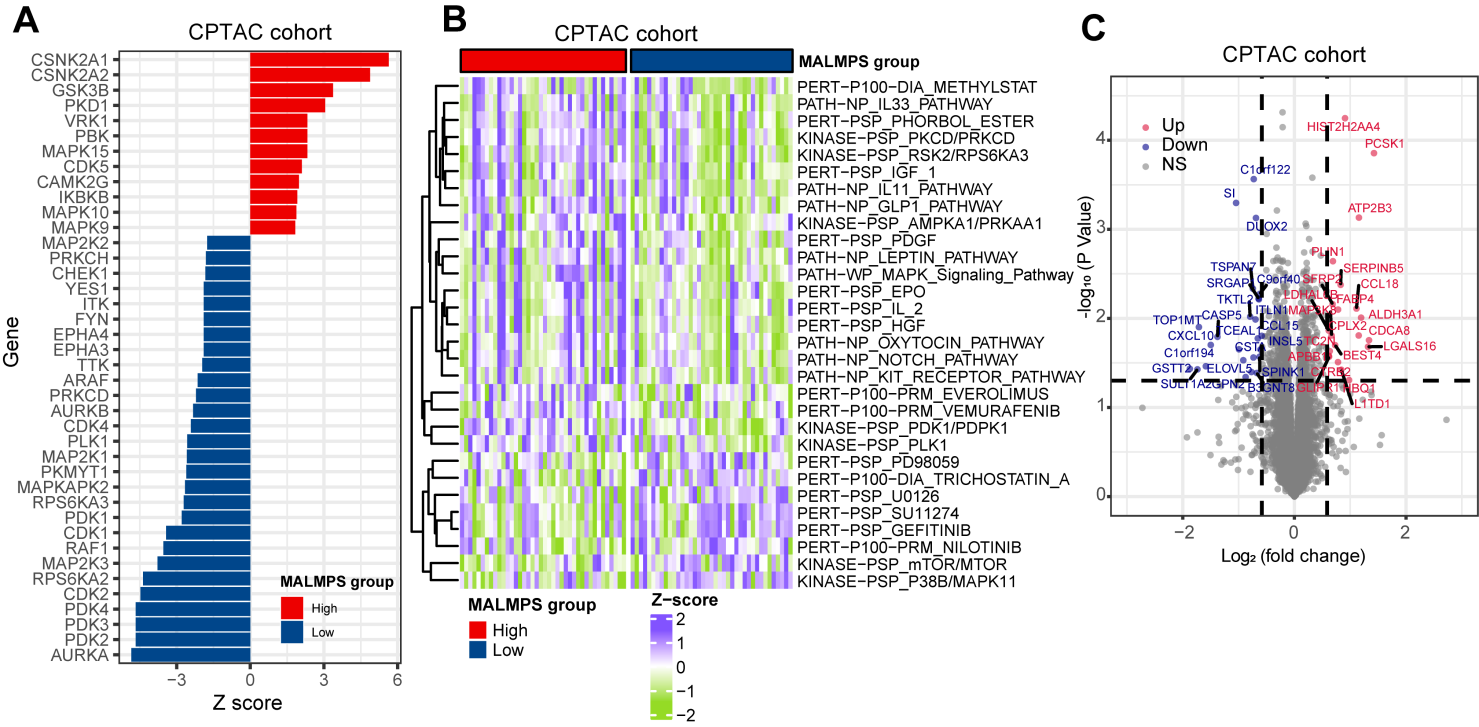


**Figure S6.** (A) Top 40 enriched kinases in high- and low-risk groups using KSEA with a significance of *P* < 0.05. (B) Heatmap showing the representative biological pathways between high- and low-risk groups in phosphoprotein level of CPTAC cohort. (C) Volcano plot of differential protein analysis between different MALMPS risk groups.

**
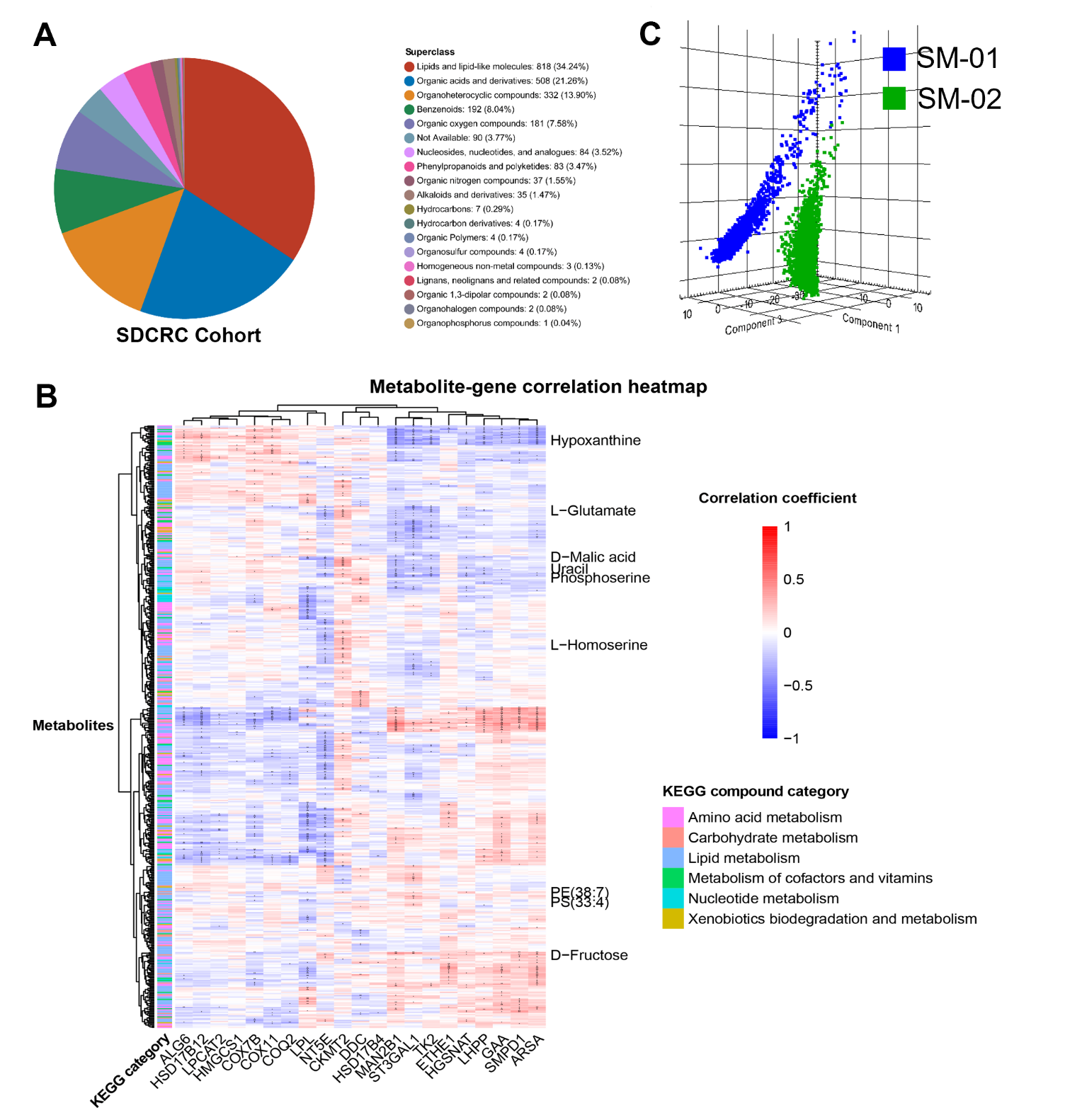
**

**Figure S7. Metabolomic and spatial metabolomic in SDCRC dataset.** (A) Pie chart showing the HMDB category of the LC-MS/MS detected metabolites in SDCRC dataset. (B) Heatmap showing the Spearman correlation of genes in MALMPS and metabolites with annotation of KEGG category. * means *P* < 0.05, ** means *P* < 0.01. (C) Principle component analysis (PCA) of spatial metabolites data showed the remarkable segmentation in samples of different MALMPS subgroup.
